# Scale‐free dynamics of core‐periphery topography

**DOI:** 10.1002/hbm.26187

**Published:** 2022-12-29

**Authors:** Philipp Klar, Yasir Çatal, Robert Langner, Zirui Huang, Georg Northoff

**Affiliations:** ^1^ Medical Faculty, C. & O. Vogt‐Institute for Brain Research Heinrich Heine University of Düsseldorf Düsseldorf Germany; ^2^ The Royal's Institute of Mental Health Research & University of Ottawa. Brain and Mind Research Institute, Centre for Neural Dynamics, Faculty of Medicine University of Ottawa Ottawa Ontario Canada; ^3^ Institute of Systems Neuroscience Heinrich Heine University Dusseldorf Dusseldorf Germany; ^4^ Institute of Neuroscience and Medicine Brain & Behaviour (INM‐7), Research Centre Jülich Jülich Germany; ^5^ Department of Anesthesiology University of Michigan Medical School Ann Arbor Michigan USA; ^6^ Center for Consciousness Science University of Michigan Medical School Ann Arbor Michigan USA; ^7^ Centre for Cognition and Brain Disorders Hangzhou Normal University Hangzhou Zhejiang China

**Keywords:** cerebral cortex topography, input processing, periodicity, pink noise, power‐law, spontaneous activity

## Abstract

The human brain's cerebral cortex exhibits a topographic division into higher‐order transmodal core and lower‐order unimodal periphery regions. While timescales between the core and periphery region diverge, features of their power spectra, especially scale‐free dynamics during resting‐state and their mdulation in task states, remain unclear. To answer this question, we investigated the ~1/*f*‐like pink noise manifestation of scale‐free dynamics in the core‐periphery topography during rest and task states applying infra‐slow inter‐trial intervals up to 1 min falling inside the BOLD's infra‐slow frequency band. The results demonstrate (1) higher resting‐state power‐law exponent (PLE) in the core compared to the periphery region; (2) significant PLE increases in task across the core and periphery regions; and (3) task‐related PLE increases likely followed the task's atypically low event rates, namely the task's periodicity (inter‐trial interval = 52–60 s; 0.016–0.019 Hz). A computational model and a replication dataset that used similar infra‐slow inter‐trial intervals provide further support for our main findings. Altogether, the results show that scale‐free dynamics differentiate core and periphery regions in the resting‐state and mediate task‐related effects.

## Graphical abstract

Scale‐free dynamics are investigated in the cerebral cortex's core‐periphery division in fMRI. Both rest and task states were assessed. We demonstrate that the brain's scale‐free dynamics are modulated by the task's periodicity in the infra‐slow band.

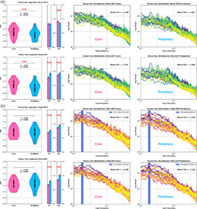

## 1 INTRODUCTION

The human brain's cerebral cortex exhibits a topography divideable into higher‐order transmodal core and lower‐order unimodal periphery regions (Bassett et al., 2008; Borgatti & Everett, 2000; Golesorkhi et al., 2021; Gollo et al., 2015; Gollo et al., 2017; Huntenburg et al., 2018; Margulies et al., 2016; Wang et al., 2019). Transmodal core or association cortices, such as the default‐mode, frontoparietal, and cingulo‐opercular networks, comprise longer intrinsic neuronal timescales with more power in relatively slower frequencies (de Pasquale et al., 2012; de Pasquale et al., 2018; van den Heuvel & Sporns, 2013). Intrinsic neuronal timescales refer to wavelengths of the brain's ongoing intrinsic activity, independent of task‐evoked activity, that can be measured by the length of the autocorrelation function or window (Wolff et al., 2022). Unimodal periphery regions include the visual, auditory, and somatomotor (SMN) cortices exhibiting shorter intrinsic neuronal timescales where power is shifted towards relatively faster frequencies compared to transmodal core regions (Bassett et al., 2008; Borgatti & Everett, 2000; Golesorkhi et al., 2021; Gollo et al., 2015; Gollo et al., 2017; Huntenburg et al., 2018; Margulies et al., 2016; Wang et al., 2019). Intrinsic neuronal timescales are preferably measured by the length of the autocorrelation function or window (ACW) (Golesorkhi et al., 2021; Ito et al., 2020; Wolff et al., 2022) where longer and more powerful timescales (wavelengths) correspond to higher ACW values and vice versa. Functional magnetic resonance imaging (fMRI) and electrophysiological EEG /MEG studies observed a higher autocorrelation in transmodal core than in unimodal periphery regions (Golesorkhi et al., 2021; Wolff et al., 2022). Paradigmatically, time‐series autocorrelation was investigated by measuring so‐called long‐range temporal correlations (LRTCs) that operate across the frequency band in electrophysiological and hemodynamic studies (He, 2014; He et al., 2010; Linkenkaer‐Hansen et al., 2001; Tagliazucchi et al., 2013). While computations in the time‐domain allow estimating the degree of temporal correlation in the recorded time‐series, as provided by the autocorrelation and the Hurst exponent (Williams, 1997; Schroeder, 2009), time‐domain computations cannot reveal the precise scaling relationship between frequency and power. Instead, scaling and scale‐free dynamics are best investigated in the log–log transformed frequency‐domain or power spectrum (Bassingthwaighte et al., 1994). These findings raise the question of how the brain's scale‐free dynamics behave in the core‐periphery topography during rest and task states. Addressing this gap in the current literature is the goal of our fMRI analysis.

Scale‐free dynamics in the frequency‐domain were frequently observed in biological systems (Szendro et al., 2001) with invariance across subjects and age in all imaging modalities, from invasive single‐unit recordings to scalp EEG and MEG, and whole‐brain fMRI (Fransson et al., 2013; Freeman et al., 2000; He, 2014; Linkenkaer‐Hansen et al., 2001) and are likely required for both healthy physiological processes in the human body including brain activity (Bassingthwaighte et al., 1994). Power spectra of the brain's spontaneous activity display scaling between frequency and power in healthy subjects under wakefulness where power logarithmically decreases as a function of frequency in fMRI (He, 2011; Huang et al., 2017; Zhang et al., 2018). On the logarithmic scale, namely in the log–log transformed frequency‐domain, BOLD power spectra display inverse power‐law distributions in the vicinity of 1/*f* pink noise where power linearly decreases as a function of frequency with a slope measured by the power‐law exponent (PLE) (He et al., 2010; Huang et al., 2017) of approximately −1 (−0.5 ≤ PLE ≤ −1.5 for pink noise [Schroeder, 2009]). The degree of the inverse power‐law distribution's slope or the level of the PLE corresponds to different noise colors, such as pink noise in fMRI under conscious wakefulness.

**FIGURE 1:**
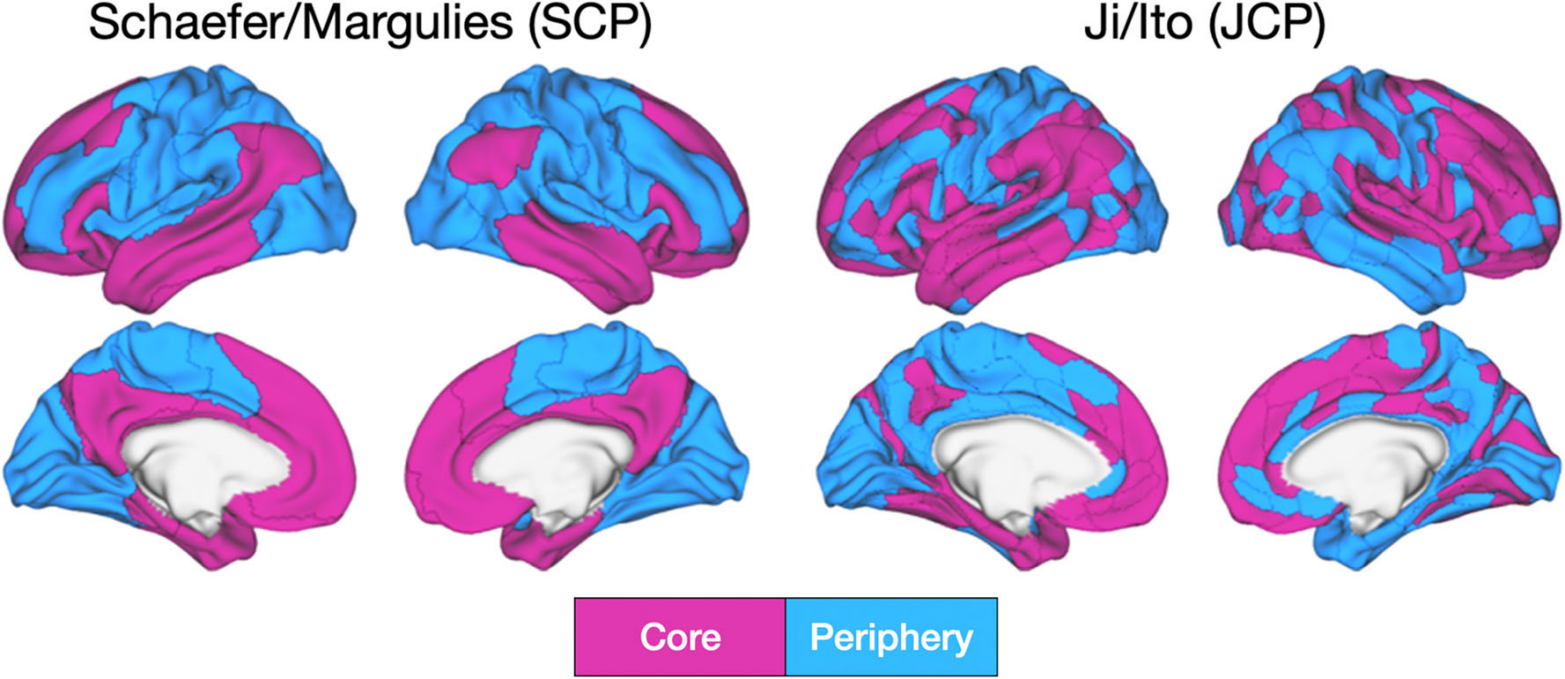
Two established templates divide the human cerebral cortex into higher‐order association cortices “core” and lower‐order sensorimotor cortices “periphery”. In the Schaefer/Margulies topography (SCP) cortical regions were derived from the Schaefer template (Schaefer et al., 2018) and divided into “transmodal core” and “unimodal periphery” ROIs based on a principal gradient that describes various cortical features (see methods part for details) (Golesorkhi et al., 2021; Huntenburg et al., 2017; Huntenburg et al., 2018; Margulies et al., 2016). The Ji/Ito (JCP) topography (Ito et al., 2020) is likewise based on a transmodal‐unimodal division from the Ji template (Ji et al., 2019). The regions were divided into core or periphery based on the transmodal‐unimodal definition in Ito et al. (2020)

Functional MRI studies assessed PLE transitions from rest to task states by applying relatively fast event‐related task designs, hence reflecting today's norm in fMRI (Huettel, 2012). Paradigmatically, He (2011) observed a widespread PLE reduction across cortical regions during a visual task relative to rest. Other studies observed a reduction of both the PLE and Hurst exponent in event‐related task designs compared to the resting‐state (Barnes et al., 2009; Churchill et al., 2016; He, 2011). Kasagi et al. (2017) showed that the task's temporal structure or periodicity significantly impacts LRTCs including the PLE in fMRI. In contrast, it remains an open question if and to what extend the BOLD's infra‐slow frequency band aligns with the corresponding infra‐slow band of environmental stimuli or tasks in the core‐periphery topography. The here utilized fMRI task design offers a unique possibility to investigate precisely this question: infra‐slow inter‐trial intervals (ITI = 52–60 s; 0.016–0.019 Hz) inside the BOLD's frequency band allow testing whether scale‐free dynamics shift power more towards slower frequencies in very slow task periodicities across the core‐periphery topography. The here presented fMRI analysis investigated scale‐free dynamics in both rest and task states focusing on the cortical core‐periphery topography that is featured by higher‐order transmodal association and lower‐order unimodal sensorimotor cortices. Two established topographical templates from Schaefer/Margulies (Margulies et al., 2016) and Ji/Ito (Ito et al., 2020) subsequently abbreviated SCP and JCP, were utilized to assess the cerebral cortex core‐periphery topography shown in Figure 1. Scale‐free dynamics were operationalized by the slope of log–log transformed power spectra, namely the PLE.

To further validate the re‐organization of power in task versus rest, measured by the level of the PLE, we additionally computed the mean frequency (MF) (Çatal et al., 2022; Golesorkhi et al., 2022). The MF is a first‐order moment statistic well‐defined for power‐law distributions with exponents ≥2. However, fMRI recordings often show lower exponents in the range of ~0.5–1 (He, 2014; Huang et al., 2017). While the inverse power‐law slope is computed on a logarithmic scale, we computed the MF in a frequency band with lower and upper limits (0.01–0.5 Hz) of the power spectrum on normal (non‐logarithmic) scale preventing the mean going up or down to infinity (in case of theoretically infinite scaling). Even though the MF is generally a moment statistic, the MF nonetheless indexes the balance between slower and faster frequency power in the power spectrum. Hence, the MF should systematically vary with the PLE in core and periphery regions, including increases or decreases from rest to task states. More precisely, PLE task increases should correspond to MF decreases and vice versa. We investigated this hypothesis by individually comparing PLE and MF rest‐task differences for all subjects (see Section 3: PLE and MF rest‐task differences) and by additionally computing the PLE‐MF correlation on a voxel‐based level (see Figure S3 in supplement). Consequently, we do not imply measuring scale‐free dynamics themselves via the MF. Instead, the MF only represents further validation of different power distributions, as the previously applied median frequency in fMRI studies (Çatal et al., 2022; Golesorkhi et al., 2022).

Our study comprised three specific aims. Aim one was the investigation of scale‐free dynamics in the core‐periphery topography during the resting‐state. Based on findings that showed longer intrinsic neuronal timescales in higher‐order association cortices (Golesorkhi et al., 2021; Wolff et al., 2022), we predicted higher PLE and lower MF levels for the core relative to the periphery regions.

Aim two examined if and to what degree the brain's scale‐free dynamics in the core‐periphery topography change during a task comprising infra‐slow frequency task periodicity. The task design included auditory self‐versus non‐self‐related trials that lasted 2 s each and were separated by very long infra‐slow frequency inter‐trial intervals (ITI) ranging from 52 to 60 s (frequency band = 0.016–0.019 Hz) (Huang et al., 2017). These long ITI allowed the proper assessment of scale‐free dynamics as they provided sufficient time to include the undershoot duration (Boynton et al., 1996; Yacoub et al., 2006) with its return to the baseline level of the brain's ongoing spontaneous activity (Huang et al., 2017). We predicted PLE increases and MF decreases in task states relative to rest based on the task's infra‐slow periodicity.

Aim three investigated to what extent the task's atypically slow event rates, namely the task's periodicity (ITI = 52–60 s; 0.016–0.019 Hz), modulates potential PLE increases from rest to task states. Besides applying several control measurements for our PLE and MF findings, we used the IRASA method to check wherever the observed power‐law distributions conformed to a scale‐free nature. Subsequently, we computed a simulation model for various degrees of extrinsic input periodicity as a further computational check for the brain's/model's PLE and MF reactivity. In addition to empirical and computational control measurements focusing on our primary dataset, we assessed a second replication fMRI dataset that offered inter‐trial intervals from 15.5 to 25.5 s corresponding to the frequency band 0.039–0.064 Hz. This second dataset replicated the core‐periphery differences and rest‐to‐task transitions of the PLE and MF measurements.

In summary, we first demonstrate individual degrees of scale‐free dynamics between core and periphery regions in the resting‐state: the core region showed higher PLE and lower MF levels than the periphery region. Second, we observed significant PLE increases and MF decreases in core and periphery regions during task states. Especially the unimodal periphery region showed significantly higher PLE increases during the task compared to the transmodal core region. Moreover, resting‐state PLE and MF differences between the core and periphery disappeared during the task's infra‐slow frequency periodicity.

## 2 METHODS AND MATERIALS

### 2.1 Subjects

We re‐used data from 25 right‐handed adults (male/female: 13/12; age 20–29 years) from a previous study (Huang et al., 2017). Participants had no history of psychiatric or neurological disorders as confirmed by a standard MRI safety screening questionnaire. Two participants were excluded due to severe head motion during fMRI scanning. Before scanning, participants answered a questionnaire with 360 questions about personal experiences regarding hobbies, birthdays, and places visited as well as simple, generally known facts. Of these, 120 questions were selected randomly for each participant (after excluding difficult questions; see Huang et al. (2017) for details). Sixty questions were randomly selected per yes/no category. Each of the questions was digitally recorded into an audio clip lasting 2 seconds by the same experimenter, and was presented once during the fMRI scan session.

### 2.2 Resting‐state

Before the task run, a 6 min resting‐state run (356 volumes) was recorded in the original study (Huang et al., 2017). Subjects were instructed to relax, stay awake, and keep their eyes closes during the scan. The brain's spontaneous activity (resting‐state) served as a baseline for comparison with task‐evoked effects on post‐stimulus activity in the task run. This allowed multiple kinds of rest‐task comparisons.

### 2.3 Task design

The analyzed dataset stems from Huang et al. (2017), and a previous study in China (Huang et al., 2014) developed the task design. The subjects had to fill out a questionnaire including 360 questions about self‐referential (autobiographical) information, such as hobbies, birthdays, places visited, and simple facts. All questions contained 6–12 Chinese syllables. A five‐point rating scale (1 denoting easiest and 5 denoting hardest) was used to estimate each question's difficulty. Questions with a score of 3 or higher were considered difficult and excluded to minimize the task's difficulty. Finally, after excluding the difficult questions, 120 questions were randomly selected for each subject. As difficult questions made up a very small proportion across all subjects, there was a sufficient number of questions with a score of <3 for each subject. Half of the questions required a “Yes” response, and the other half a “No” response. Each question was digitally recorded into an audio clip lasting 2 s by the same experimenter and was presented once during the fMRI scan session.

We analyzed one task run with a length of 20 min (1184 volumes). The task design comprised 20 trials separated by very long ITI varying unpredictably from 52 to 60 s, jittered in 2 s steps. The ITI range corresponded to the infra‐slow frequency range of 0.016–0.019 Hz. The 20 trials were assigned pseudorandomly to the 120 questions, and each trial's auditory stimulus time lasted 2 s. In addition to assessing the BOLD signal evoked by the trials, such long ITIs provide sufficient time for the undershoot (Boynton et al., 1996; Yacoub et al., 2006) and the return to the baseline level of brain activity (see figure 1 in Huang et al. (2017)). The long ITIs additionally avoided potential nonlinearities caused by overlapping hemodynamic responses between succeeding trials (Fox et al., 2006). During trials, participants were instructed to press a left or right button of a response box with their right‐hand index or middle finger to indicate a “yes” or “no” answer to the auditory stimuli presented. To ensure participant cooperation and alertness, responses were monitored during the experiment. Stimulus presentation and response recording were done using E‐Prime (Psychology Software Tools, Pittsburgh, PA), and stimuli were delivered via an audiovisual stimulus presentation system designed for an MRI environment. The volume of the headphones was adjusted to the comfort level of each subject.

### 2.4 MRI data acquisition

A GE 3 T (Discovery MR750) scanner with a standard head 8‐channel head coil was used to acquire gradient‐echo echo‐planar imaging (EPI) images of the whole brain (time repetition 1.0 s; time echo 25 ms; 21 slices; slice thickness = 6 mm; no inter‐slice gap; spacing = 0; field of view = 210 mm^2^; flip angle = 76°; image matrix: 64 × 64). The resting‐state comprised with 360 volumes (6 min); the task run comprised 1184 volumes (19:44 min). During each EPI scan, subjects were instructed to relax, stay awake, and keep their eyes closed. Eye‐tracking during fMRI was not available, but off‐line post‐scan recordings ensured that subjects complied with this instruction. Cardiac and respiratory physiological signals were recorded for resting‐state and task runs.

### 2.5 fMRI data preprocessing

Preprocessing was performed using AFNI (https://afni.nimh.nih.gov) (Cox, 1996) applying the following steps: (1) removing the first four volumes of each fMRI run; (2) physiological noise regression using time‐locked cardiac and respiratory signals using AFNI's RETROICOR; (3) slice timing correction; (4) rigid body realignment using AFNI's 3dvolreg for volume‐wise realignment of estimated head motion parameters. Subjects exhibiting more than 5% censored volumes in the resting‐state run were excluded from further data analysis. Volumes with head motion shifts >0.3 mm or rotation >3° were censored in both rest and task runs; (5) co‐registration with high‐resolution anatomical images; (6) initial spatial normalization of the anatomical scans into MNI152 2009c stereotactic space and subsequent functional to anatomical alignment (normalization); (7) functional resampling to 3 × 3 × 3mm^3^ voxels; (8) regression of linear and nonlinear drift (equivalent to a high‐pass filtering of 0.0067 Hz), average eroded white matter signal (WM) and CSF to reduce non‐neuronal noise (Jo et al., 2013); and (9) spatial smoothing using an 8 mm full‐width at half‐maximum isotropic Gaussian kernel.

### 2.6 Analysis of PLE and MF in a replication fMRI dataset

A second clinical dataset of subjects undergoing an elective transsphenoidal approach for resection of a pituitary microadenoma from Huang et al. (2018) served to replicate the findings of our analysis. The sample comprised 20 right‐handed participants (male/female: 8/12; age 32–64 years) under conscious wakefulness. Analyses of subjects in sedated and anesthetic states were not included. Six subjects were removed due to low volume quality and a high level of head motion. We utilized an 8 min eyes‐closed resting‐state (240 volumes) and an 18 min auditory task with comprising self and non‐self‐related trials (569 volumes) from the wakefulness session (TR = 2 s). A sparse event‐related design with ITI ranging from 15.5 to 25.5 s, jittered by 2 s steps, was adopted. The task's temporal range corresponds to the infra‐slow frequency range of 0.039–0.064 Hz. Sixty trials (30 self‐related; 30 non‐self‐related), lasting for 0.5 s each, were presented (Huang et al., 2018).

### 2.7 Templates for the core‐periphery topography

Two established templates for the cerebral cortex's core‐periphery topography were adopted. For template one (Schaefer/Margulies) (Margulies et al., 2016), subsequently abbreviated “SCP”, cortical regions were derived from the Schaefer atlas (Schaefer et al., 2018). The second template stems from Ji/Ito (Ito et al., 2020), subsequently abbreviated “JCP.” The SCP template comprises 200 and the JCP template 360 cortical regions. The SCP template was divided into seven networks: visual, SMN, dorsal attention (DAN), salience, limbic, frontoparietal (FPN), and default‐mode network (DMN). The JCP template offers 12 networks: primary visual 1 (V1), primary visual 2 (V2), auditory (AUD), SMN, cingulo‐opercular (CON), DMN, dorsal attention (DAN), frontoparietal cognitive control (FPN), posterior multimodal (PMM), ventral multimodal (VMM), orbito‐affective (ORA), and language (LAN).

The SCP and JCP templates were divided into higher‐order transmodal association and lower‐order unimodal sensorimotor ROIs based on the first principal gradient presented in (Golesorkhi et al., 2021; Huntenburg et al., 2017; Huntenburg et al., 2018; Margulies et al., 2016). This gradient measured the variance of cortical properties, such as functional and structural connectivity, cytoarchitecture, myeloarchitecture, gene expression, and the length the brain's intrinsic neuronal timescales (Baldassano et al., 2017; Huntenburg et al., 2018; Margulies et al., 2016). These studies demonstrated a moderate relationship between the similarity of the aforementioned gradient's properties between two or more regions and the region position along the cortical surface. Following this gradient and previous analyses (Golesorkhi et al., 2022), the regions were assigned to either “core” or “periphery” ROIs as follows. (1) SCP ROIs: based on the networking assignment and first principal gradient (Margulies et al., 2016), limbic, FPC, and DMN regions were included into core, while visual, SMN, dorsal attention, and salience regions were assigned to the periphery category. (2) JCP ROIs: based on the Ji/Ito template and the unimodal‐transmodal distinction (Ito et al., 2020), DAN, PMM, VMM, ORA, LAN, CON, FPN, DMN were assigned to the core region. The periphery regions comprised V1, V2, AUD, and SMN networks.

It is noteworthy that the dorsal attention network (DAN) belongs to the periphery region for the SCP ROI, whereas the DAN belongs to the core region for the JCP ROI. The attribution of regions to either core or periphery for the SCP ROI is based on the first principal gradient in Margulies et al. (2016). In the article by Margulies et al. (2016), Figure 3b shows the DAN region's wide extension across unimodal sensory regions, such as the visual and SMN cortices, and across heteromodal association regions, such as the frontoparietal and salience networks. The attribution of the DAN to the periphery (SCP) and core (JCP) thus presents an attempt to balance the DAN region's impact on the core versus periphery PLE levels.

### 2.8 Power‐law exponent (PLE) analysis

Increasing frequencies go along with decreasing power following a power‐law function of $P=\frac{1}{f^{\beta }}$ where f is frequency, P is power, and the β is the PLE (Bak et al., 1987; Schroeder, 2009). Applying the logarithm on the frequency‐domain, that is, log(f) and log(P), reveals inverse power‐law distributions. The slope determined by a linear regression using least‐square estimation between log(f) and log(P) is the PLE. The computation of the PLE was performed as follows. First, AFNI's 3dPeriodogram transformed the time‐series into the frequency‐domain on a voxel‐based level. The power spectra were cut to the frequency band 0.01–0.5 Hz. The lower frequency limit was chosen to avoid signal contributions from scanner drift (Fransson et al., 2013), whereas the higher limit was constrained by the sampling rate (Nyquist frequency). For each subject the ROI‐based (average) log–log transformed power spectra were extracted and fitted with a linear least‐square regression.

### 2.9 PLE control analysis I: IRASA method

To discard the possibility of the oscillatory component of the power spectrum driving our results, we used irregular‐resampling auto‐spectral analysis (IRASA) method to separate fractal component from oscillatory component (Muthukumaraswamy & Liley, 2018; Wainio‐Theberge et al., 2021; Wainio‐Theberge et al., 2022; Wen & Liu, 2016). Briefly, the signal is resampled with a factor h ranging from 1.1 to 1.9 with steps of 0.05; and $\frac{1}{h}$. Geometric means for of up and downsampled PSDs were computed, then the median of power of the geometric means across different h values was defined as the scale‐free component. The intuition behind the method is that the scale‐free method is resilient against resampling whereas oscillatory component is not (Wen & Liu, 2016). PLE values were computed as the slope of the linear regression fit to log‐power and log‐frequency, but in the range of 0.01–0.25 Hz since downsampling reduces the Nyquist frequency. PLE values from the IRASA method were compared with the conventional PLE results (recalculated in 0.01–0.25 Hz range) using *t*‐tests.

### 2.10 PLE control analysis II: Comparison with surrogate data

To test the goodness of fit for scale‐invariance, we adapted a goodness of fit test for power law distributions (Clauset et al., 2009) which was used in fMRI studies (Çatal et al., 2022; He, 2011; Scalabrini et al., 2017; Tagliazucchi et al., 2013). For each ROI in rest and task, 1000 time series of fractional Gaussian noise (fGN) with the same length, standard deviation and Hurst exponent as averaged time series of real data were simulated (Stoev et al., 2006). fGN is a model of stationary scale‐free dynamics (Beran, 1994). Power laws were fitted to each of the PSDs of synthetic time series and real data using the maximum‐likelihood estimation method (Clauset et al., 2009). We used Kolmogorov–Smirnov distance D to measure the goodness of fit: the larger the D, worse the fit. *p*‐value was defined as the fraction of synthetic time series with Ds that are larger than the D of the real data. The hypothesis that the fMRI signal is scale‐free was ruled out if *p* < .05.

### 2.11 Additional PLE control analyses

Besides the IRASA method and comparison with surrogate data, we applied five additional control analyses for rest and task PLE using the primary and replication datasets. The detailed results are presented in the supplement.

- Two time windows (volumes 199–555 and 549–905) of the task time‐series were matched to the resting‐state length of 356 volumes. PLE and MF were computed for both windows to control that higher task PLE values were not a result of the task run's longer length (compared to the resting‐state run). Figures S1 and S2, including Table S1, in the supplement, show the results.
- PLE and MF were computed and correlated on a voxel‐based level for each subject in all ROIs, including rest and task states, to assess the systematic relationship between PLE and MF. The voxel‐based PLE‐MF analysis allowed checking wherever the ROI‐based averages also hold and correlate on the voxel level in rest and task states, respectively. Figure S3 and Table S2 in the supplement show the results.
- We individually computed time windows for single‐trial PLE and MF (each window = 2 s trial plus 52 s post‐stimulus activity) for self‐related and non‐self‐related trials. Albeit in a limited frequency range, this allowed controlling if the task effect on PLE and MF holds irrespective of the task's cognitive kind or content (self or non‐self). Figure S4 and Table S3 in the supplement show the results.
- Several studies demonstrated variance of cortical features along a gradient, such as for functional and structural connectivity, cytoarchitecture, myeloarchitecture, gene expression, and the length (plus power) of the brain's intrinsic neural timescales (Baldassano et al., 2017; Huntenburg et al., 2018; Margulies et al., 2016). We computed the PLE and MF for all single ROIs that constitute the SCP topography to control that PLE and MF changes in response to the task were manifest across the core‐periphery topography. Figure S5 in the supplement shows the results.
- Besides applying motion correction in the preprocessing, we extracted all six estimated head motion time‐series (three translational and three rotational motion parameters). The head motion time‐series were then transformed into the log–log frequency‐domain to compute the head motion PLE. This allowed us to correlate the BOLD's PLE for all ROIs in rest and task states with the estimated head motion PLE (Scalabrini et al., 2019) to exclude the possibility that BOLD PLE results were significantly affected by the subjects' head motion during functional scanning. Table S5 in the supplement shows the results.

### 2.12 Correlation between task PLE and reaction times

Our fMRI analysis' third aim was the investigation to what extent the task's infra‐slow event rates (ITI = 52–60 s; 0.016–0.019 Hz) can explain the potential PLE increase from rest to task states. It requires consideration that PLE changes in task states can also rest on cognitive factors from the subjects' ongoing experience. Paradigmatically, subjects may undergo expectational or attentional differences during task runs that affect the BOLD's power distribution, including the PLE, that diverges from the resting‐state. Therefore, we correlated the task's PLE for all ROIs with the subjects' reaction times. More precisely, we applied the Pearson and Spearman correlation for self‐ and non‐self‐related trials with the ROI‐based PLE values to check wherever the PLE in task states correlated with the subjects' reaction times. A substantial modulation of PLE changes in task states by cognitive processes such as attention is unlikely if a lack of significant correlations occurs across all ROIs.

### 2.13 Mean frequency (MF) analysis

Computation of MF used AFNI's 3dPeriodogram to compute the power spectrum on a voxel‐based level. The same frequency band (0.01–0.5 Hz) as for the PLE computation was applied. In a second step, AFNI's 3dTstat was used to compute both (1) the sum of power multiplied by frequency and (2) the sum of power. The power times frequency is then divided by the power. Consequently, only the MF per voxel remains. In difference to the PLE, we computed the MF on normal (non‐logarithmic) scale in the limited frequency band of 0.01 to 0.5 Hz. The limited frequency band prevents the MF from going up or down to infinity in case of theoretically infinite scaling.

### 2.14 Calculations of PLE and MF rest‐task differences

Beside core versus periphery comparisons that we individually assessed in resting‐state and task, we additionally calculated three further comparisons that statistically compared rest versus task states. The comparisons between rest and task states followed aim two of our analysis, namely to examine to what degree the brain's scale‐free dynamics change or align during the task's infra‐slow periodicity. We computed these three calculations for both ROIs (SCP and JCP) using the subjects' ROI‐based mean values. Furthermore, the three calculations were individually applied for both measurements (PLE and MF) and we labeled them: (1) Intra‐ROI Rest‐Task Difference, (2) Inter‐ROI Rest‐Task Difference, and (3) Core‐Periphery Difference. We consequently describe the methodological approach for all three additional calculations.

- Intra‐ROI rest‐task difference: The first calculation analyzed wherever the observed rest‐to‐task PLE and MF changes were statistically significant. We individually performed this calculation for the core and periphery regions. The results subsequently allowed checking the statistical significance via paired t‐tests of rest versus task PLE and MF changes. We repeated the same calculation for the periphery region.
- Inter‐ROI rest‐task difference: The second calculation first subtracted rest from task values individually for the PLE and MF within each region. The results first revealed the absolute PLE and MF changes within the core and periphery regions. In a second step, we statistically compared the absolute change between core and periphery using a paired *t*‐test for PLE and MF. We accordingly labeled this calculation “Inter‐ROI rest‐task difference” to test wherever the periphery region exhibits a superior reactivity or alignment in task states compared to the core region.
- Core‐periphery difference: The third calculation first subtracted periphery from core values, individually for resting‐state and task. In a second step, using paired t‐tests we statistically compared the rest (core minus periphery) versus task (core minus periphery) results. This calculation allowed testing wherever topographical differences of the PLE and MF observed in the resting‐state significantly changed in task states.

### 2.15 Simulation of task periodicity in an extended frequency range

A simulation model was applied to further investigate the effect of the task's frequency in an extended frequency range. Five thousand instances of colored noise with a power spectral density of P=1fβ where f is frequency, β is the slope of the power spectrum and P is the power of that respective frequency were simulated with MATLAB's dsp.ColoredNoise function from DSP System Toolbox. dsp.ColoredNoise uses an algorithm described in Kasdin (1995). Briefly, Gaussian white noise is colored by multiplication by an autoregressive model of order 63 in the frequency domain. β values were pseudorandomly chosen from a uniform distribution between −0.5 and −1.5 to be consistent with the empirical data observed across the individual subjects. PLE and MF values were calculated in the simulated noises. In a second step, to simulate the effect of the task, sine waves with three different frequencies, 0.016 Hz (low), 0.18 Hz (mid), and 0.45 Hz (high) representing task periodicity were generated and added to the original noises. These sine waves were added to the original colored noises and PLE and MF values were again calculated. To see the effect of task periodicity on the change in PLE (and MF), the change in those measures after the addition of the sine wave was calculated.

## 3 RESULTS

### 3.1 PLE in resting‐state

The PLE values of both core regions (SCP = −1.108; JCP = −1.101) were significantly higher than in the periphery regions (SCP = −1.021, *t* = −5.71, *p* < .001; JCP = −1.008, *t* = −5.97, *p* < .001). Plotted on log–log graph paper, the resting‐state power spectra showed a linear decrease as a function of frequency, that is, typical inverse power‐law distributions for brain dynamics in the range of pink noise (Gisiger, 2001; He, 2014; He et al., 2010). We additionally computed the coefficient of variation (CV) for core and periphery regions. A higher compared to a lower CV can represent the capacity for better input processing (Golesorkhi et al., 2021; Zilio et al., 2021). The CV was significantly higher (*p* < .001) in the periphery only for the JCP ROI (core = −0.156, periphery = −0.184), whereas the CV for the SCP ROI (core = −0.154, periphery = −0.171) yielded no statistical significance (*p* = .945).

### 3.2 PLE in task

**FIGURE 2:**
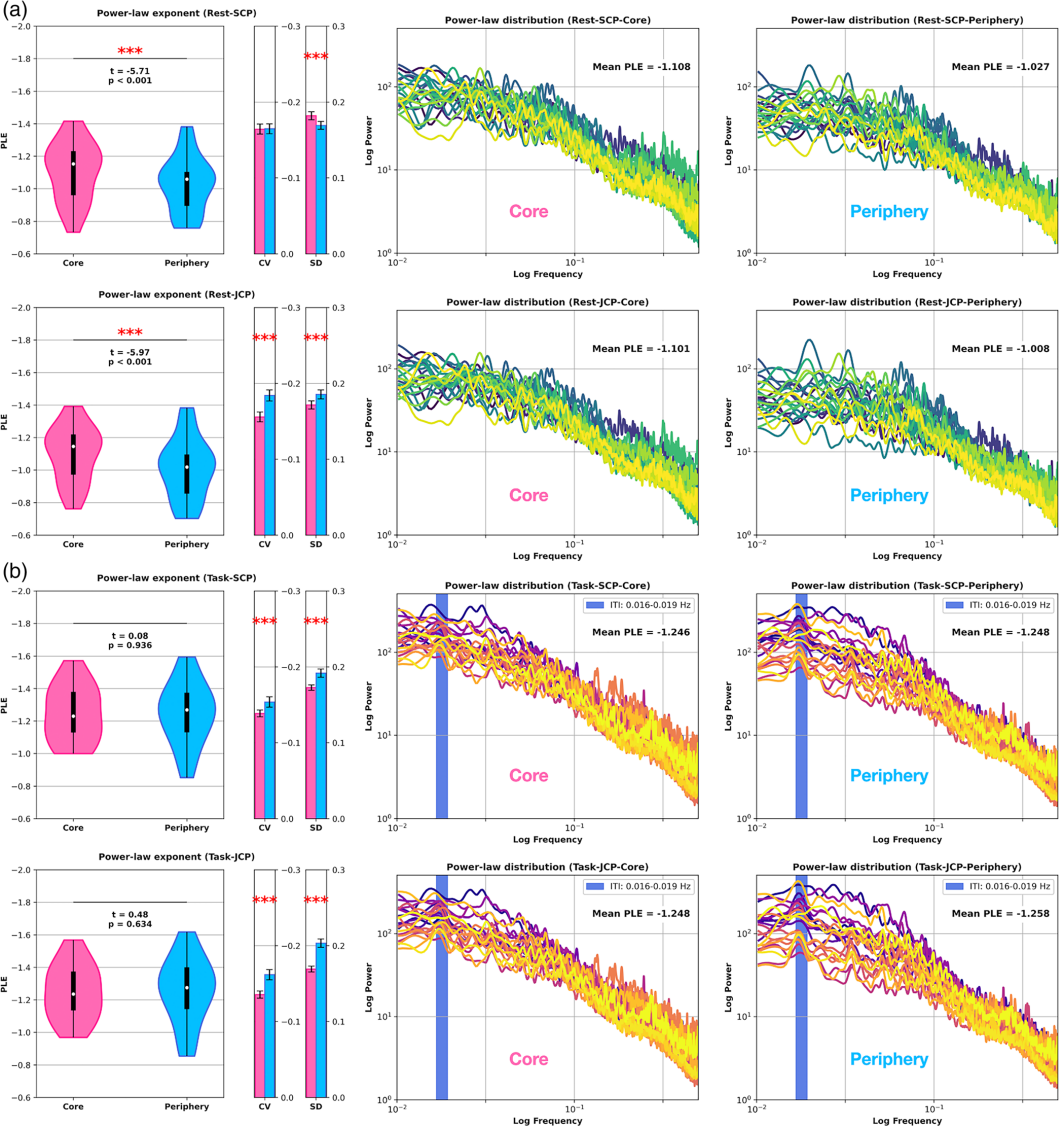
Inverse power‐law distributions and PLE where each line represents one subject. **a)** SCP (row one) and JCP (row two) resting‐state. The core‐periphery comparison yielded significant PLE differences for both ROIs. **b)** SCP (row one) and JCP (row two) task. The PLE significantly increased and converged between core and periphery for the SCP and JCP ROIs in task states. Vertical bars in the task log–log power spectra represent the inter‐trial interval (52–60 s; 0.016–0.019 Hz). CV, coefficient of variation; PLE, power‐law exponent; SD, standard deviation

Significant core‐periphery PLE differences, as observed in the resting‐state, completely vanished during task states. The PLE values between core and periphery converged for both ROIs (SCP: *t* = 0.08, *p* = .936; JCP: *t* = 0.48, *p* = .634). Compared to the resting‐state, PLE in both core (SCP = −1.281; JCP = −1.248) and periphery PLE (SCP = −1.271; JCP = −1.258) significantly increased. In contrast to the PLE, the inter‐subject CV of the PLE decreased in both core and periphery regions during task (compared to the resting‐state). The CV was significantly higher (*p* < .001) in the periphery for both ROIs (SCP = −0.148; JCP = −0.162) compared to the core (SCP = −0.134; JCP = −0.135). Figure 2 displays the resting‐state and task power‐laws, where each line represents an individual subject, including the mean PLE across subjects.

### 3.3 Correlation between task PLE and reaction times

The mean reaction time for self‐related trials across subjects was 2.39 s (SD = 0.298; CV = 0.125), and 2.76 s for non‐self‐related trials (SD = 0.445; CV = 0.162). Faster reaction times to self‐ versus non‐self‐related trials were expected based on a previous EEG study (Kolvoort et al., 2020) investigating scale‐free dynamics, including self‐ versus non‐self‐related processing.

The correlations between the task's PLE results for all ROIs and the subjects' reaction times to self‐ and non‐self‐related trials turned out non‐significant. The *p*‐values for the Pearson correlation were all above *p* ≥ .1775, whereas the *p*‐values for the Spearman correlation were all above *p* ≥ .1096 without applying corrections for multiple comparisons. Additionally, both Pearson's correlation coefficient and Spearman's rho showed much higher values between the task's PLE levels in all four ROIs (SCP and JCP core‐periphery) and the non‐self‐related trials (as compared to self‐related trials). These observations indicate that observed PLE increases in task states were unlikely primary driven by attentional processes or a cognitive preparation towards the occurrence of the trials, precisely because one would expect a higher correlation between the PLE and self‐related trials, instead of the observed higher correlation between PLE and non‐self‐related trials. The detailed results are shown in Figure S6 and Table S6.

### 3.4 MF in resting‐state

The MF in both periphery regions (SCP = 0.14; JCP = 0.14) was significantly higher than in the corresponding core regions (SCP = 0.132, *t* = −6.24, *p* < .001; JCP = 0.133, *t* = −4.94, *p* < .001). This indicates that periphery regions contained more power in faster relative to slower frequencies compared to the core regions. Inter‐subject CV of MF values showed the same pattern as the CV of PLE, being significantly higher (*p* < .001) in the periphery regions (SCP = 0.118; JCP = 0.123) compared to the corresponding core regions (SCP = 0.102; JCP = 0.094).

### 3.5 MF in task

**FIGURE 3:**
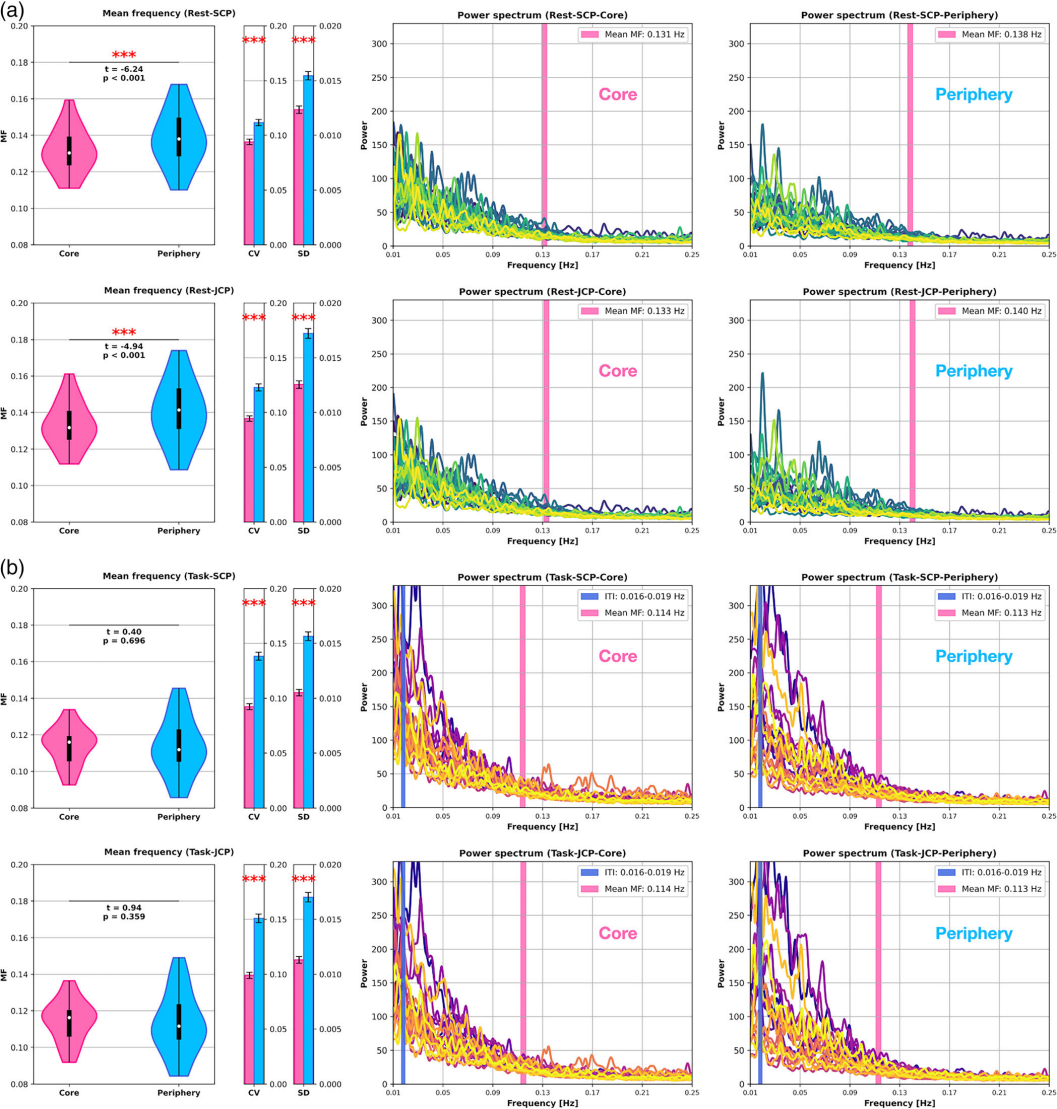
Power spectra and MF where each line represents one subject. (a) SCP (row one) and JCP (row two) resting‐state. The core‐periphery comparison yielded significant MF differences for both ROIs. (b) SCP (row one) and JCP (row two) task. In task, MF significantly decreased and converged between core and periphery for both the SCP and JCP ROIs. Vertical bars in the power spectra represent the MF and inter‐trial interval (52–60 s; 0.016–0.019 Hz). CV, coefficient of variation; MF, mean frequency; SD, standard deviation

In contrast to the resting‐state MF and in accordance with the PLE task results, the core‐periphery comparison no longer yielded a significant difference in task states. Both MF core (SCP = 0.112; JCP = 0.114) and MF periphery (SCP = 0.112; JCP = 0.113) decreased to the same level, respectively (SCP: *t* = 0.4, *p* = .696; JCP: *t* = 0.94, *p* = .359). Consequently, the task state induced a shift of power towards slower frequencies compared to the initial resting‐state power distribution. The inter‐subject CV values slightly increased compared to the resting‐state except for SCP Core. MF resting‐state and task results are displayed in Figure 3 where each line represents an individual subject. Table 1 provides an overview of both PLE and MF results in resting‐state and task.

**TABLE 1:** PLE and MF core‐periphery comparison

Computations	Run	ROI	Core	Periphery	*t*‐value	*p*‐value
PLE	Rest	SCP	−1.108 (−0.154/0.173)	−1.021 (−0.171/0.174)	−5.71	*p* < .001
		JCP	−1.101 (−0.156/0.172)	−1.008 (−0.184/0.186)	−5.97	*p* < .001
	Task	SCP	−1.281 (−0.134/0.172)	−1.271 (−0.148/0.186)	0.08	.936
		JCP	−1.248 (−0.136/0.17)	−1.258 (−0.162/0.203)	0.48	.634
MF	Rest	SCP	0.132 (0.102/0.013)	0.14 (0.118/0.016)	−6.24	*p* < 0.001
		JCP	0.133 (0.094/0.013)	0.1402 (0.123/0.017)	−4.94	*p* < .001
	Task	SCP	0.112 (0.098/0.011)	0.112 (0.144/0.016)	0.40	.696
		JCP	0.114 (0.099/0.011)	0.113 (0.151/0.017)	0.94	.359

*Note*: Data represents mean values including their respective coefficient of variation (CV) (first value or left in bracket) and standard deviation (SD) (second or right value in bracket). MF, mean frequency; PLE, power‐law exponent; statistics, student's paired *t*‐test.

### 3.6 Additional PLE and MF control analyses results

We applied several control analyses using the primary and replication datasets to validate the PLE and MF results. First, we computed the PLE and MF using two time windows of the task time‐series matched to the resting‐state length. The two time‐matched task windows allowed checking the possibility that PLE increases and MF decrease in the full‐length task, as compared to the resting‐state, resulting in the task's higher length. Both the PLE and the MF task results observed in the time‐matched task windows matched the full‐length task run and are displayed in Figures S1 and S2, including Table S1, in the supplement.

Second, we computed and correlated PLE and MF on a voxel‐based level in addition to the ROI‐based level. The systematic relationship between PLE and MF obtained for the ROI‐based level did hold on the voxel‐based level, showing high correlations in all ROIs (*r* ≥ 0.951). Figure S3 and Table S2 in the supplement display the results. The voxel‐based PLE results in Figure 3 also highlight that voxel‐based PLE values range from white noise (PLE = ~ 0) over pink noise (PLE = −1) to brown noise (PLE = −2). Hence, it is important to consider that the observed pink noise in the core‐periphery topography during rest and task states represents an ROI‐based average, while the voxel‐based BOLD signal spans across a mutual continuum of PLE levels from white to brown noise.

Third, individual time windows for single‐trial PLE and MF (each window = 2 s trial plus 52 s post‐stimulus activity) for self‐related and non‐self‐related trials showed no statistically significant differences. Neither the PLE nor the MF results varied between self‐ versus non‐self‐related time windows, suggesting that PLE and MF changes were not dependent on the task's cognitive kind or content.

Fourth, we computed single ROI (SCP) PLE and MF results to control that the PLE increased and that MF decreased from rest to task states in all single ROIs as observed in the core‐periphery topography. The single ROI results, presented in Figure S5 and Table S4, followed the same rest‐to‐task changes that we observed in the core‐periphery topography.

Fifth, the six head motion parameters' PLE levels did not substantially correlate with the BOLD's PLE shown in Table S5. This result implies the independence of the BOLD's PLE from head motion effects.

### 3.7 PLE rest‐task differences

Besides the presented core versus periphery comparisons that we individually assessed in rest and task states above, we calculated three additional comparisons (see the method part for details on the three additional PLE and MF calculations). The three additional PLE and MF calculations followed aim two of our analysis, namely the comparison between rest and task states to investigate the brain's reactivity or alignment with the task's infra‐slow periodicity. We subsequently present the PLE results, whereas the MF results follow in the section below.

**FIGURE 4:**
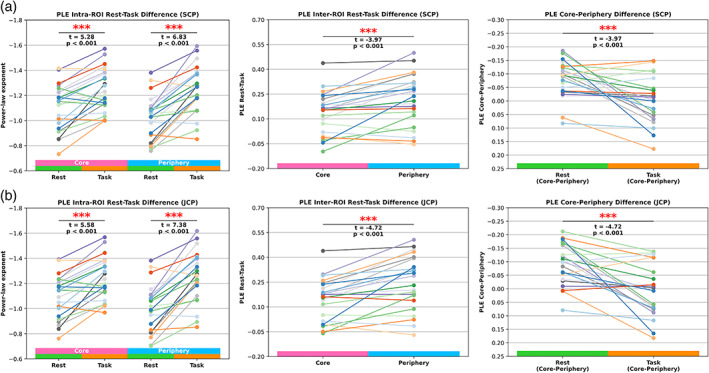
Three additional PLE comparisons where each line represents one subject. (a) SCP ROI results. The left plot (intra‐ROI rest‐task difference) shows the PLE increase from rest to task states for all subjects in core and periphery, respectively. The middle plot (inter‐ROI rest‐task difference) shows task values subtracted from rest values, respectively for core and periphery. The right plot (core‐periphery difference) displays the results of the core minus periphery calculations for rest and task, respectively. (b) JCP ROI results for the same three PLE comparisons

(1) Intra‐ROI rest‐task difference: The PLE significantly increased from resting‐state to task for the core regions (SCP: *t* = 5.28, *p* < .001; JCP: *t* = 5.58, *p* < .001). The periphery regions showed an even higher PLE increase in response to task states (SCP: *t* = 6.83, *p* < .001; JCP: *t* = 6.83, *p* < .001). Consequently, the periphery regions reacted or aligned best to the task by showing the highest PLE and MF changes compared to the core regions.

(2) Inter‐ROI rest‐task difference: The highest differences were observed for the periphery (SCP: PLE = 0.221; JCP: PLE = 0.25) and lower absolute differences for the core regions (SCP: PLE = 0.139; JCP: PLE = 0.147). These results were significant for both SCP (*t* = −3.97, *p* < .001) and JCP (*t* = −4.72, *p* < .001) ROIs and highlight that, conceived in absolute rest minus task values, the periphery regions exhibited a stronger alignment with the task compared to the core regions.

(3) Core‐periphery difference: The statistical comparison between core minus periphery (rest) and core minus periphery (task) yielded significant results (SCP: *t* = 3.97, *p* < .001; JCP: *t* = −4.72, *p* < .001). These results highlight that the topographical differences of the PLE observed in the resting‐state significantly diminished in task states. Figure 4 displays the additional three calculations.

### 3.8 MF rest‐task differences

The three additional comparisons for the MF follow subsequently.

**FIGURE 5:**
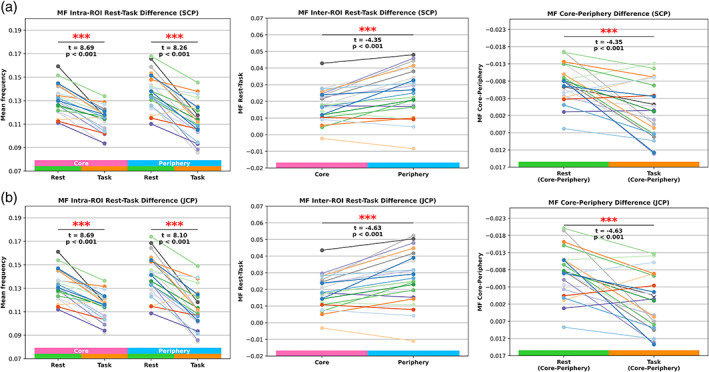
Three additional MF calculations where each line represents one subject. (a) SCP ROI results. The left plot (intra‐ROI rest‐task difference) shows the MF decreases from rest to task states for all subjects in core and periphery, respectively. The middle plot (inter‐ROI rest‐task differences) shows task values subtracted from rest values, respectively for core and periphery. The right plot (core‐periphery difference) displays the results of the core minus periphery calculations for rest and task, respectively. (b) JCP ROI results for the same three MF comparisons

(1) Intra‐ROI rest‐task difference: MF values significantly decreased from resting‐state to task for core (SCP: *t* = 8.69, *p* < .001; JCP: 8.69, *p* < .001) and periphery (SCP: *t* = 8.26, *p* < .001; JCP: *t* = 8.1, *p* < .001) regions. Decreasing MF values in task states following observed PLE increases in task states. A shift of power from faster to slower frequencies increases the inverse power‐law distributions' slope (PLE) so that moment statistics, such as the mean, also shift to relatively slower frequencies.

(2) Inter‐ROI rest‐task difference: The periphery regions yielded the highest differences, and the comparison with the core regions yielded statistical significance (SCP: *t* = −4.35, *p* < .001; JCP: *t* = −4.63, *p* < .001). Therefore, absolute MF decreases to slower frequency power in response to task states were higher in the periphery than in the core region.

(3) Core‐periphery difference: The statistical comparison between core minus periphery (rest) and core minus periphery (task) yielded significant results (SCP: *t* = −4.35, *p* < .001; JCP: *t* = −4.63, *p* < .001). As observed for the PLE, topographical differences of MF prevalent in the resting‐state dissolved in task states. Figure 5 displays the results of the three additional MF calculations. Table 2 summarizes the three additional PLE and MF calculations.

**TABLE 2:** Intra‐ROI rest‐task difference, inter‐ROI rest‐task difference, and core‐periphery difference

Computations	ROIs	Rest	Task	*t*‐value	*p*‐value
**Intra‐ROI rest‐task difference**					
PLE	SCP Core	−1.108	−1.246	5.276	*p* < .001
	SCP Periphery	−1.027	−1.248	6.834	*p* < .001
	JCP Core	−1.101	−1.248	5.578	*p* < .001
	JCP Periphery	−1.008	−1.258	7.378	*p* < .001
MF	SCP Core	0.131	0.114	8.689	*p* < .001
	SCP Periphery	0.138	0.113	8.256	*p* < .001
	JCP Core	0.133	0.114	8.685	*p* < .001
	JCP Periphery	0.140	0.113	8.098	*p* < .001
Computations	ROIs	Core	Periphery	*t*‐value	*p*‐value
**Inter‐ROI rest‐task difference**					
PLE	SCP	0.139	0.221	−3.967	*p* < .001
	JCP	0.147	0.250	−4.718	*p* < .001
MF	SCP	0.017	0.025	−4.348	*p* < .001
	JCP	0.019	0.027	−4.634	*p* < .001
Computations	ROIs	Rest	Task	*t*‐value	*p*‐value
**Core‐periphery difference**					
PLE	SCP	−0.081	0.001	−3.967	*p* < .001
	JCP	−0.093	0.009	−4.718	*p* < .001
MF	SCP	−0.007	0.0006	−4.348	*p* < .001
	JCP	−0.007	0.0015	−4.634	*p* < .001

*Note*: Data represents mean values. MF, mean frequency; PLE, power‐law exponent; statistics, student's paired *t*‐test.

### 3.9 PLE control analysis I: Distinction between fractal and oscillatory components (IRASA)

**FIGURE 6:**
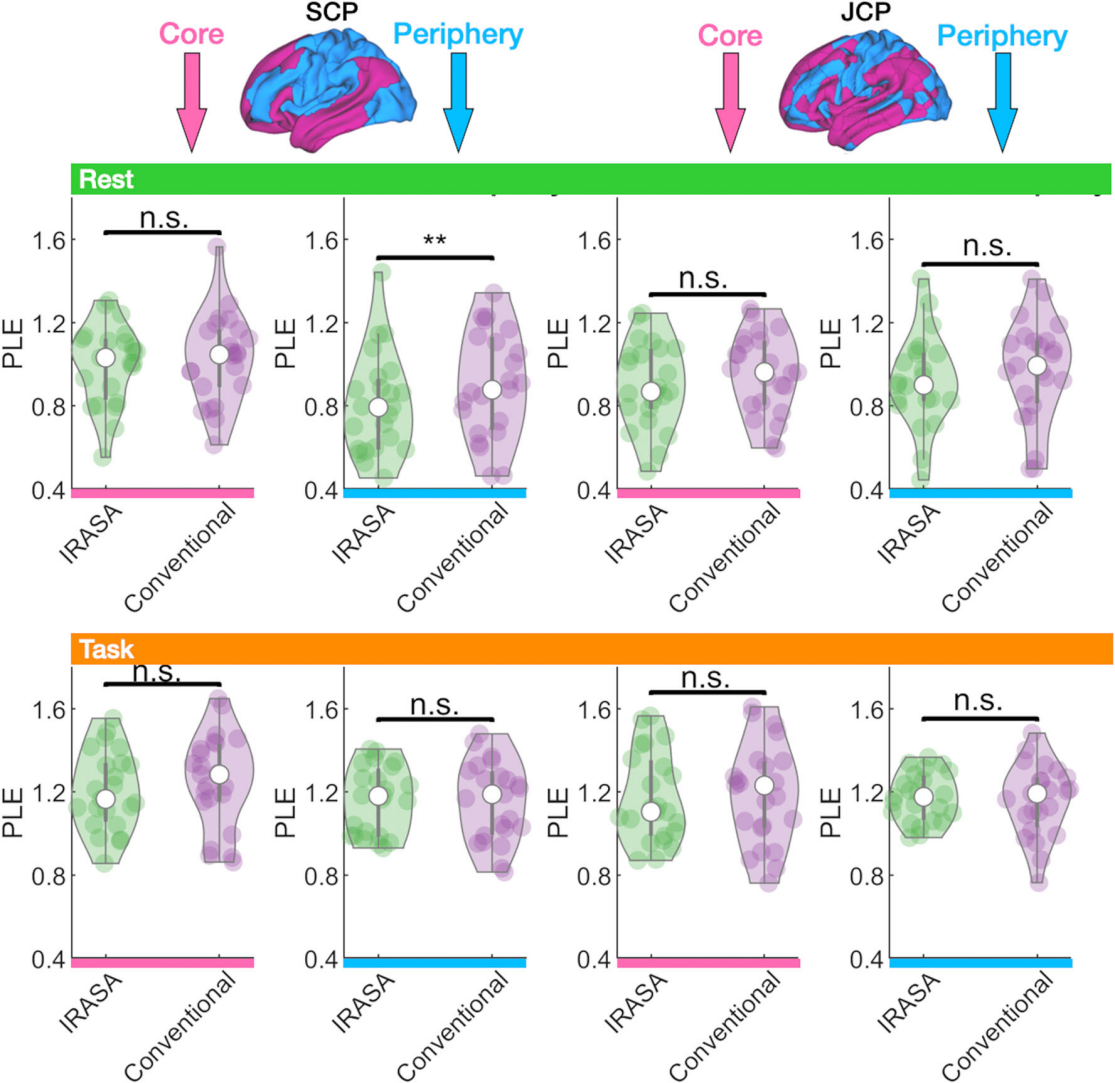
Application of the IRASA method. PLE values were calculated via the IRASA method and the conventional method for both the SCP and JCP ROIs including rest and task states. A significant difference between the IRASA and conventional method only occurred in the SCP periphery ROI

The IRASA method (Wen & Liu, 2016) was applied to separate oscillatory and fractal components of the power spectrum and previously successfully in EEG/MEG (Wainio‐Theberge et al., 2021; Wainio‐Theberge et al., 2022). The comparison between the conventionally computed PLE presented above, including both fractal and oscillatory components in the power spectrum, and the IRASA method obtained fractal‐based PLE values (exclusion of oscillatory components) for the SCP and JCP ROIs in rest and task states are displayed in Figure 6. The comparison between both analysis methods yielded no significant differences. The IRASA results indicate that observed PLE increases in task states were not driven by oscillatory components. Instead, the IRASA results demonstrate that the observed PLE increases in task reflect a genuine change of the brain's fractal or scale‐free dynamics, that is, a real change of the power‐law distribution's slope across the different frequency bands rather than a change in only a particular oscillatory frequency related to the task's infra‐slow frequency or periodicity.

### 3.10 PLE control analysis II: Comparison with surrogate data

In addition to the IRASA method, we further tested the suggested scale‐free dynamics of our data by comparing the goodness of fit of the power‐law to the PSDs of real data and simulated fractional Gaussian noise (fGN) (Çatal et al., 2022; Clauset et al., 2009; He, 2011; Scalabrini et al., 2017; Tagliazucchi et al., 2013). *p*‐values were obtained for all ROIs. This implies that the fraction of synthetic time‐series had a significantly worse fit than the real data. The *p*‐values for all ROIs exceeded .1, except for the SCP Periphery in Task (*p* = .057). Except for this ROI, the results show that the fraction of synthetic time‐series had a significantly worse fit compared to the empirical data. The results can be found in Table S7.

### 3.11 PLE and MF results in the replication dataset

Besides analyses in our primary dataset, we assessed the core versus periphery PLE and MF differences, including the variables rest‐to‐task transitions, in a second replication dataset (Huang et al., 2018). This dataset offered a comparable task design that, like our primary dataset, included self and non‐self‐related trials with shorter yet long ITI ranging from 15.5–25.5 s (0.039–0.064 Hz). All PLE (Figure S7) and MF (Figure S8) results in the core‐periphery topography, including their rest versus task changes, obtained in the primary dataset were successfully replicated in the same SCP and JCP ROIs (see also Table S5 for an overview of PLE and MF results).

It is noteworthy that we observed lower PLE levels in the replication dataset in rest (PLE ~ −0.8) and task (PLE ~ −0.89) states compared to our primary dataset (rest PLE ~ −1.05; task PLE ~ −1.24). Two possible reasons may account for different absolute PLE levels between both datasets.

One possible reason is that the primary dataset subjects' age spanned from 20 to 29 years, whereas the replication dataset clinical subjects' ages spanned from 32 to 64 years. Churchill et al. (2016) investigated scale‐free dynamics in fMRI via the Hurst exponent, a measure of LRTCs in the time‐series. They compared two groups of younger subjects (20–33 years, median age 24) versus older subjects (61–82 years, median age 68) concerning aging effects on fractal scaling. In short, Churchill et al. (2016) observed that the older group showed higher decreases in the Hurst exponent across task states in several brain regions compared to the younger group. Another fMRI study (Dong et al., 2018) also investigated scale‐free dynamics via the Hurst exponent in 116 healthy subjects (19–85 years, median age 43) and observed increases as well as decreases of the Hurst exponent in different brain regions, hence also underlining the effect of age on scale‐free dynamics.

Another possibility is that impacts on the power‐law distributions and PLE can stem from different MRI scanners, including diverging functional scan settings between the primary and replication datasets. Paradigmatically, the primary dataset used a repetition time (TR) of 1 s, whereas the second replication dataset TR was 2 s. Regarding the repetition time, Mikkelsen and Lund (2013) demonstrated that the scaling index, in their analysis assessed via the Hurst exponent, is sensitive to the sampling rate (time repetition) in fMRI BOLD. We must interpret the absolute PLE and Hurst exponent levels in fMRI with caution and focus on the relative differences between regions, including relative (percentage) changes from rest to task states that we compared between the two datasets ROIs in Table 3. The percentage rest‐to‐task PLE increase between the primary and replication datasets shows a higher PLE increase in the primary dataset that comprised longer ITI. When comparing the percentage PLE increases between both datasets, we found an approximately ~60–71% higher PLE increase in the primary dataset with longer ITI (52–60 s) compared to the replication dataset with shorter ITI (15.5–25.5 s). This comparison between both datasets showed a stable relationship of the percentage rest‐to‐task PLE increases within a range of 11%.

**TABLE 3:** Relative or percentage PLE increase from rest to task states in a comparison between both datasets

ROI	Primary dataset (ITI = 52–60 s; 0.016–0.019 Hz)	Replication dataset (ITI = 15.5–25.5 s; 0.039–0.064 Hz)	Percentage increase of replication dataset compared to. Primary dataset
SCP core	12.52	7.73	61.74
JCP core	13.37	9.55	68.47
SCP periphery	21.50	14.72	71.43
JCP periphery	24.82	14.96	60.27

*Note*: Data represents the intra‐ROI percentage rest to task PLE increase in columns two (primary dataset) and three (replication dataset) of the Table. The last column shows the percentage of the replication dataset's task PLE increase compared to the primary dataset's PLE increase.

### 3.12 Simulation of task periodicity in an extended frequency range

**FIGURE 7:**
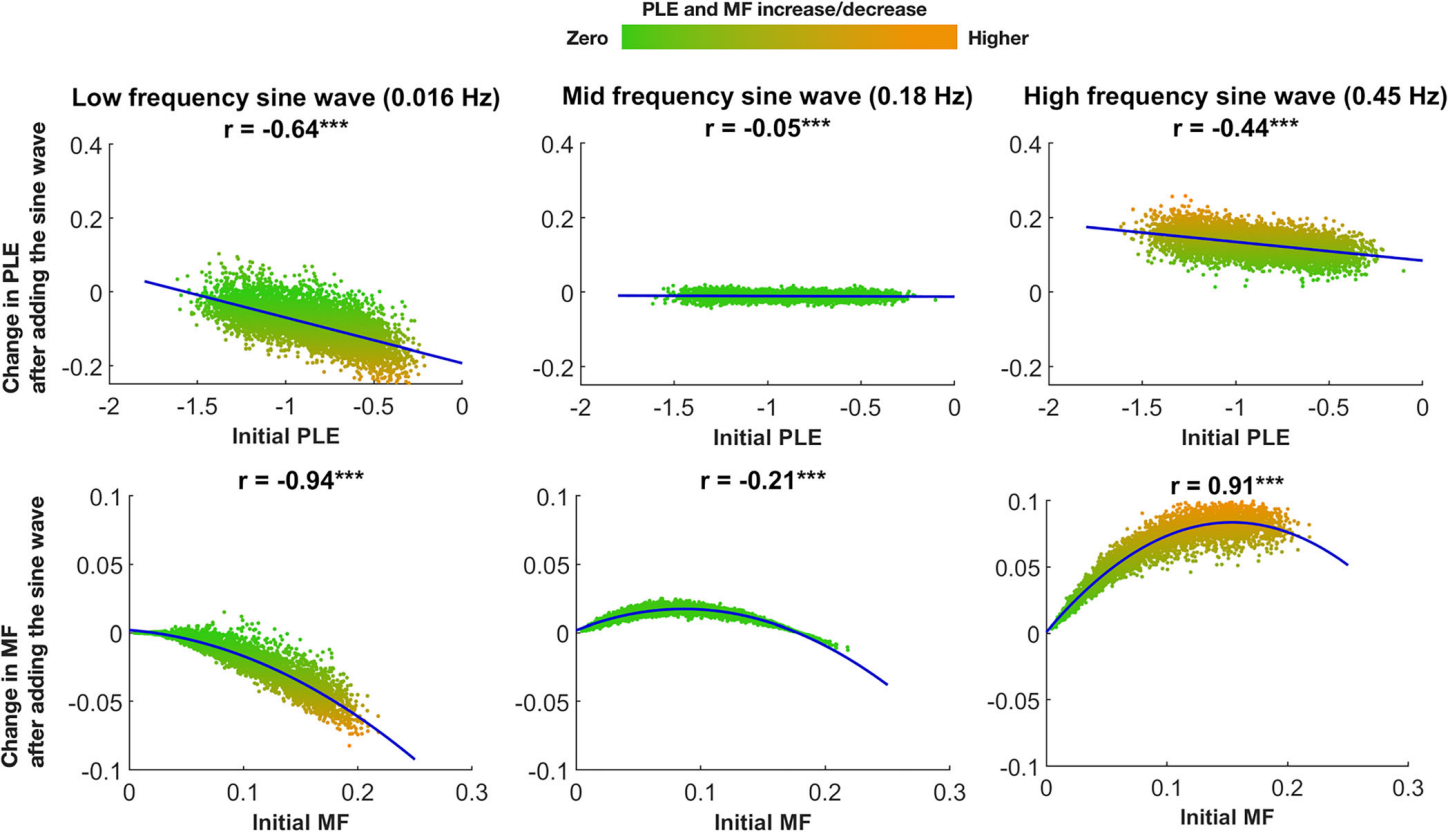
Computational model simulating three levels of task periodicity. We generated 5000 instances of colored noise (represented by the dots in each plot) and added sine waves with frequencies of 0.016, 0.18, and 0.45 Hz. We calculated the PLE and MF values before and after simulating the three levels of sine wave frequencies. The top row shows a continuum of initial PLE values before adding the sine waves (*x*‐axis) and the PLE change after adding the sine waves (*y*‐axis). The bottom row shows the same continuum and change for the MF

To assess the relationship between the task's frequency and its effect on the PLE and MF, we generated 5000 instances of colored noise, and sine waves of frequencies 0.016, 0.18, and 0.45 Hz were added (see Methods). We simulated 5000 instances of colored noise and three levels of sine wave frequencies (0.016, 0.18, and 0.45 Hz) to model the impact of the task's frequency (ITI) on the PLE and MF (see the methods section for details). The slow wave frequency of 0.016 Hz increased the slope of the PLE (more negative slope) and decreased the MF, consistent with the empirical findings from both datasets. The mid‐frequency oscillatory sine wave of 0.18 Hz caused a minor impact on the model. Finally, the high‐frequency sine wave of 0.45 Hz decreased the slope of the PLE and increased the MF, consistent with the shorter ITI of other studies (He et al., 2010; Kasagi et al., 2017; Lin et al., 2016). We used Pearson's correlation for the PLE (r‐values are shown in Figure 7). For MF, due to the nonlinearity of the relationship between initial and task‐evoked MF changes, we used Spearman correlation and fitted second‐order polynomials, shown in Figure 7. Of note, the change in PLE and MF can be seen as a function of the initial value in the low and high‐frequency sine waves, whereas this does not seem to be the case in mid‐frequency. Figure 7 displays the model's results.

## 4 DISCUSSION

### 4.1 Scale‐free dynamics diverged between core and periphery regions in the resting‐state

Aim one of our fMRI analysis was the investigation of resting‐state scale‐free dynamics in the cerebral cortex's core‐periphery topography. The resting‐state provided a baseline for possible PLE and MF changes in task states.

The core regions showed significantly higher PLE and lower MF levels, as compared to the periphery regions, in the primary and second replication datasets (see Figures S7 and S8 for the replication results). Higher PLE levels in higher‐order transmodal association cortices converge with LRTCs and longer intrinsic neuronal timescales observed in electrophysiological EEG plus MEG (Golesorkhi et al., 2021; Wolff et al., 2022) and fMRI studies of the same regions (Stephens et al., 2013; Yeshurun et al., 2021). Consequently, the observed PLE levels in the frequency‐domain in both core and periphery regions correspond to resting‐state scale‐free dynamics found in the time‐ and frequency‐domain by other studies (Ciuciu et al., 2012; Ciuciu et al., 2014; Rocca et al., 2018; Roisen & Shew, 2020).

The BOLD's relatively higher power in slow frequencies observed in the core compared to the periphery regions reflects higher temporal stability or autocorrelation of the signal in higher‐order transmodal cortices (Shew et al., 2015; Shew & Plenz, 2013; Tagliazucchi et al., 2012). High‐power and slow‐frequency timescales naturally carry information via longer temporal correlations over extended periods, providing long‐range memory effects in ongoing brain activity (He, 2011; Linkenkaer‐Hansen et al., 2001; Meisel et al., 2017; Sporns, 2011; Tagliazucchi et al., 2013; Wolff et al., 2022). A theoretical inference based on the PLE and MF results is that the core regions may exhibit a “robustness against rapid change” induced by ongoing intero‐ and exteroceptive input streams from the body and the environment. This robustness appeared to be particularly strong in the core region, whereas the robustness was lower in the periphery, possibly due to the periphery's need for continuous sensitivity to sensory stimuli.

Inspecting the inverse power‐law distributions in Figure 2 revealed the periphery regions' higher inter‐subject PLE variability, especially in slower frequencies, compared to the core regions. The periphery regions' higher inter‐subject variability showed statistically significant results in the JCP ROI's standard deviation and CV in the primary dataset's resting‐state. In the replication dataset's resting‐state, the periphery regions showed statistically significant higher standard deviation and CV results in both the SCP and JCP ROIs.

Conversely, the core regions showed more uniform power‐law distributions between subjects (see especially the slower frequencies in Figure 2), as mirrored in the SCP and JCP ROIs' CV and SD in the main and replication datasets, with the only exception being the main dataset's SCP ROI showing a higher SD in the core region. This observation might be in accordance with the idea that core regions exhibit a “robustness against rapid change” to preserve the signal's identity and higher autocorrelation over longer timescales. Low‐power high‐frequency fluctuations are nested within high‐power and low‐frequency timescales, allowing the brain to differentiate itself from ongoing extrinsic perturbations, notably in the core region. The periphery regions defined the other side of this mutual spectrum: the periphery may lack the robustness of powerful longer timescales, whereas the periphery regions provide a higher “space of possibilities” amounting to higher degrees of freedom towards quickly aligning with extrinsic demands of the environment (Bertschinger & Natschläger, 2004; Kello et al., 2010; Linkenkaer‐Hansen et al., 2001; Poil et al., 2008).

### 4.2 Scale‐free dynamics converged between core and periphery regions in task states

Aim one provided the ground for aim two, namely the investigation of whether brain activity shifts its power away from faster frequencies towards slower frequencies, given a task paradigm with an atypical inter‐trial interval (ITI) that falls inside the infra‐slow frequency band. It is required to distinguish two questions and the associated aims two and three here. Aim two focused on investigating if substantial PLE and MF changes occur during task states relative to previously observed resting‐state levels. Another question is why the brain significantly shifted its power towards slower frequencies in task states and if this observation was related to the task's infra‐slow interval, or if cognitive processes during the task run, such as minder‐wandering or attention, primarily impacted the PLE increase in task states. This question refers to aim three tackled in the next section. We subsequently elaborate on aim two.

Interestingly, the significant division between core and periphery regions concerning PLE and MF measurements, as previously observed in the resting‐state, dissolved below statistical significance in task states in the primary and replication datasets. We replicated the PLE increase and MF decrease in task states and their respective convergence between the core and periphery regions via computing two task time windows. Both time windows were time‐matched to the resting‐state length to rule out the possibility that the task's longer run impacted the PLE increase and core‐periphery convergence (see Figures [Link], [Link], and Table S1). Therefore, the degree of scale‐free dynamics measured by the PLE thus converged across the cerebral cortex showing a holistic PLE increase in task states. We excluded the possibility that the same PLE and MF levels in task states resulted from single ROIs with extreme rest‐to‐task increases by analyzing PLE and MF transitions from rest‐to‐task states in all ROIs that constituted the SCP core‐periphery topography. We observed significant PLE increases and MF decreases across all single ROIs (see Figure S5 and Table S4).

Concerning the convergence between the core and periphery regions' PLE and MF levels in task states, we must remember that the SCP and JCP core regions showed higher resting‐state PLE levels. Consequently, periphery regions showed relatively higher PLE increases and MF decreases in the rest‐to‐task transition compared to core regions shown in Figures 4 and 5 in the Section 3. Conceivable are two theoretical possibilities for the periphery regions' relatively higher PLE increase and MF decrease in task states: (1) faster and less powerful timescales are naturally associated with shorter lifetimes as faster timescales decay more rapidly. Consequently, sensory inputs from the environment can imprint themselves more strongly in the periphery regions, where exteroceptive inputs substantially shift the brain's power distribution following the periphery's larger “space of possibilities” for a brain‐environment alignment, as mentioned in the previous part (Heiney et al., 2021; Kello et al., 2010). (2) Another theoretical possibility is that the task's infra‐slow frequency range better matches or corresponds to the core regions' default (or resting‐state) infra‐slow BOLD dynamics. Consequently, task‐evoked re‐distributions of power across the frequency band turn out lower in the core than in periphery regions, possibly because the core's pre‐stimulus dynamics or activity variability already matches the task's dynamics to a better degree, hence providing a form of “preadaptation” by the core regions.

### 4.3 Modulation of scale‐free dynamics by the task's temporal structure

Aim two uncovered wherever substantial changes of scale‐free dynamics occur in task states relative to rest states. Aim three focused to what extent the task's atypically slow event rates themselves, provided by the task's infra‐slow periodicity in the primary dataset (ITI = 52–60 s; 0.016–0.019 Hz) and the replication dataset (ITI = 15.5–25.5 s; 0.039–0.064 Hz), modulated the significant PLE increase from rest to task states.

The PLE increase in task states represents a surprising finding since previous EEG, MEG, ECoG (He et al., 2010; Lin et al., 2016), and fMRI (He et al., 2010; Kasagi et al., 2017) often observed PLE decreases in relatively fast event‐related and block task designs. Conversely, our fMRI analysis with atypically long ITI found substantial PLE increases in task states across the cerebral cortex in two core‐periphery topographies (SCP and JCP) in the primary and replication datasets. A straightforward inference to the task's ITI as a primary modulatory factor for task‐related PLE increases is flawed, since cognitive processes, such as mind‐wandering, attention, and other possible modulatory effects during the inter‐trial interval, can also account for the observed PLE increases in task states.

To control the impact of cognitive processes during the task run in the primary dataset, we correlated the subjects' reaction times in response to self‐ and non‐self‐related trials with the subjects' task PLE levels. Our reasoning for testing the impact of cognitive processes on the observed PLE increases in task states is as follows: a statistically significant correlation between reaction times and PLE, especially for self‐related trials due to the faster reaction times (mean = 2.39 s; SD = 0.29) compared to non‐self‐related trials (mean = 2.76 s; SD = 0.45), could support the hypothesis that task‐related PLE increases can also root in cognitive processes. Across all ROIs (SCP and JCP; core and periphery) the Pearson's correlation coefficient and Spearman's rho lacked statistical significance, while on average across ROIs, non‐self‐related trials showed a substantially higher correlation with the PLE in task states (Pearson's *r* = 0.24; Spearman's rho = 0.26) than self‐related trials (Pearson's *r* = 0.033; Spearman's rho = 0.078) (see Figure S6 and Table S6).

In addition to the question of wherever cognitive and other possible processes substantially modulated task‐evoked PLE increases, one could ask if self‐related versus non‐self‐related trials individually impacted the task's PLE increase. The computation of self‐related and non‐self‐related time windows demonstrated that neither the PLE nor the MF time windows, where we compared self‐related versus non‐self‐related trials against each other, yielded significant differences in any of the SCP and JCP ROIs (see Figure S4 and Table S3). These results suggested that PLE increases and MF decreases in task states are unlikely due to the task's cognitive kind or content.

Nonetheless, it requires consideration that while the correlations between task PLE and the subjects' reaction times in response to the trials (PLE‐RT correlation) turned out non‐significant, the 95% confidence intervals showed a wide range, such as from −0.29 to 0.52 for Pearson's *r* in the SCP core region (see Figure S6 and Table S6). Based on these confidence intervals and the generally low results of power tests that we applied for the correlations, we can neither rule out nor suggest significant task PLE‐RT correlations. Future studies offering specifically designed task paradigms that systematically vary the task's ITI, including neutral stimuli or tasks beyond a self‐ versus non‐self‐related dichotomy, are required to investigate task‐related PLE changes in task designs that better diminish potential impacts of the stimulus' cognitive kind, content, or demand.

Our fMRI analysis emphasizes the potential impact of the task's temporal structure or ITI beyond the task's cognitive contents or demands without negating possible contributions by cognitive modulations. We suggest that besides other potential influences discussed above and in the methodological limitations section, the task's temporal structure also modulated the brain's scale‐free dynamics by increasing their PLE in both the very slow ITI of the primary dataset (0.016–0.019 Hz) and the slow ITI of the replication dataset (0.039–0.064 Hz). The model results (Figure 7) of three sine wave frequencies supported the modulatory role of the task's temporal structure on PLE changes by demonstrating a systematic relationship between the extrinsic frequency and the PLE changes.

**FIGURE 8:**
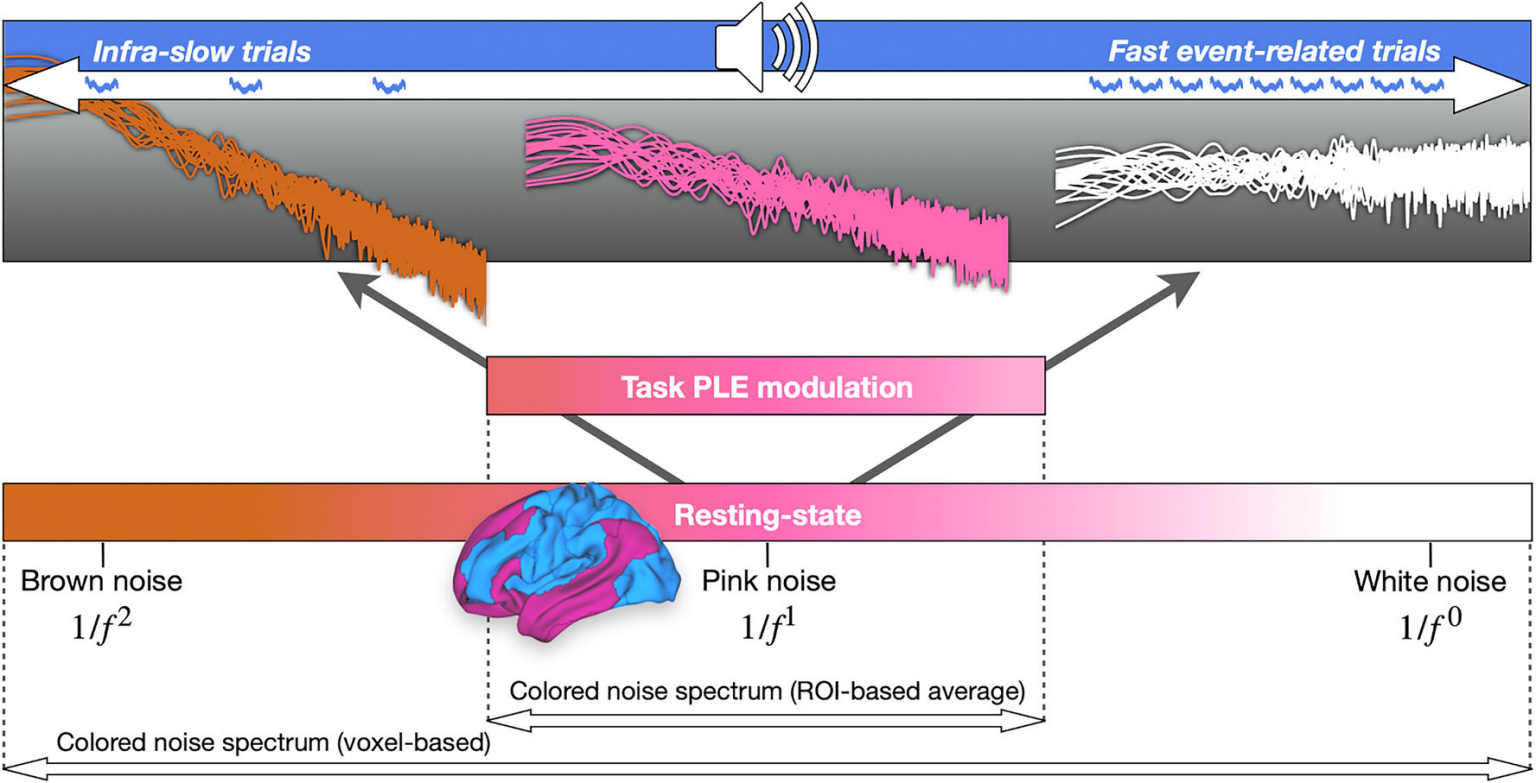
Colored noise spectrum of the power‐law exponent (PLE) in fMRI. Resting‐state recordings observed average PLE levels of −1 corresponding to pink noise. We also observed average ROI‐based PLE levels of −1 (pink noise), while voxel‐based PLE levels (see Figure S3) ranged from white noise (PLE = 0) over pink to brown noise (PLE = −2). In fast event‐related task designs, the PLE level often decreases towards white noise (see introduction and discussion). Conversely, we observed PLE increases more towards brown noise in task states with trials in the infra‐slow frequency range supporting a modulatory role of the task's inter‐trial interval on PLE changes

Taking our observations together with previous findings of scale‐free dynamics (Beggs & Plenz, 2003; Cocchi et al., 2017; Morales & Muñoz, 2021; Petermann et al., 2009; Shew et al., 2015; Shew & Plenz, 2013; Tagliazucchi et al., 2012), scale‐free dynamics appear to operate on a mutual continuum (Figure 8). This continuum spans from white, over pink, to brown noise in fMRI (see the voxel‐based PLE range in Figure S3). Several fMRI studies in the infra‐slow band (0.01–0.1 Hz) reported average PLE values between −0.4 to −1.1 (Ciuciu et al., 2012; Fransson et al., 2013; He, 2011; Huang et al., 2017). Pink noise dynamics are neither highly predictable, such as brown noise with relatively stable dynamics or minor variations in time, nor entirely unpredictable, such as white noise with high variability and lack of regularity. Instead, pink noise represents an intermediate mixture between variability and regularity. Following these findings, the brain's pink noise likely reflects relatively high sensitivity towards sensory input streams while preserving a sufficient amount of intrinsic temporal structure. We observed shifts of power more towards the slower pole of brown noise in the task state comprising slow inter‐trial intervals, whereas power shifts more towards the faster end of white noise often occurred in fast event‐related designs with shorter ITI or task states that required ongoing cognitive demands such as attention (He, 2011; He et al., 2010; Kasagi et al., 2017; Lin et al., 2016).

### 4.4 Methodological limitations

Several limitations of our fMRI analysis require consideration. Based on our empirical findings and the model's results, we suggested that the PLE can systematically vary in accordance with the task's frequency range (ITI). However, the task's ITI represents only one of several possible factors that can modulate PLE changes from rest to task states, as implicated by another fMRI analysis (He, 2011).

The fMRI study by He (2011) also compared rest versus task states, the latter with a comparable ITI of 17.3–30.2 s (frequency band = 0.033–0.058 Hz) to our replication dataset ITI of 15.5–25.5 s (0.039–0.064 Hz). Conversely to our observations of increased PLE levels in task states, the findings by He (2011) showed significant PLE decreases in various ROIs across the brain. The two conflicting results are reconcilable by considering the different nature of the task itself between the study by He (2011) and the primary and replication datasets analyzed by us. The task paradigm by He (2011) instructed subjects to report via button press when an identical crosshair occasionally changed from white to dark gray for short periods of only 250 ms. This kind of visual detection task requires the subjects' ongoing attention to the crosshair because the subjects can otherwise easily miss the slight change of the crosshair's color occurring every ~17–30 s. The task paradigms of our primary and replication datasets instructed the subjects' to perceive auditory stimuli where the headphone volume was adjusted to the comfort level for each subject individually. Hence, the subjects could not miss the auditory stimuli, reducing the load on cognitive attentional processes during the task run. Based on the considerations above and suggested by He (2011), it is feasible that brain activity shifted power towards faster frequencies in the study by He (2011) to accommodate the subjects' ongoing information processing. Conversely, a power shift towards slower frequencies could be modulated by the tasks' infra‐slow periodicity, as observed in our primary and replication datasets. Especially required are task designs constructed to precisely test the modulation of the stimuli or task inter‐trial intervals on rest‐to‐task PLE changes. In this respect, our fMRI analysis' results cannot warrant that the task's ITI primarily modulated the observed rest‐to‐task PLE increases. Instead, our fMRI analysis indicates the potential impact of the task's temporal structure on scale‐free dynamics. Future studies offering task paradigms that systematically vary the inter‐trial intervals with neutral stimuli beyond self‐ versus non‐self‐related trials are required.

Another limitation is the PLE comparison across different imaging modalities. Further fMRI, EEG, and MEG studies are required to investigate modulatory effects by various task inter‐trial intervals on scale‐free brain dynamics. Task designs that systematically vary the ITI are necessary for two reasons. First, EEG and MEG studies reported different PLE levels (Bénar et al., 2019) compared to functional MRI. Second, observed PLE values in fMRI are often lower compared to PLE values in MEG and especially in EEG (see He et al. (2010) for PLE comparisons between the imaging modalities). The physiological foundation of scale‐free dynamics, especially the observed PLE transition from rest to task states, is not established (Lin et al., 2016).

It also requires consideration that estimating scale‐free dynamics by inverse power‐laws is methodologically not straightforward, and the inference that a signal exhibits scale‐free properties can be problematic. Paradigmatically, Broido and Clauset (2019) discuss the often‐made claim that networks are scale‐free based on the estimation that the fraction of nodes follows a power‐law. The authors argue that this claim is sometimes not warranted under more stringent statistical data testing. Rather than following a power‐law, Broido and Clauset (2019) suggest that the data may better be describable by a log‐normal or a heavy‐tailed distribution that decays more slowly than an exponential. We acknowledge that we tested our observed power‐laws to the best of our knowledge, whereas the results of even more stringent tests remain unsolved and require application in future studies.

Finally, the correlations between the subjects' reaction times to the trials and the task‐evoked PLE levels turned out low and non‐significant. However, it can be interesting for future investigations to assess the probability that, in other datasets and task designs, PLE increases or decreases are possibly predictable based on the subjects' reaction times, highlighting a closer relationship between PLE changes and behavioral task performance.

## 5 CONCLUSION

This fMRI analysis investigated scale‐free dynamics in the cerebral cortex's core‐periphery topography during the resting‐state and a task paradigm with atypical slow inter‐trial intervals that fall within the BOLD's infra‐slow frequency band. We observed a significant division between the PLE and MF levels between the core and periphery regions in the resting‐state. The transmodal core regions showed higher PLE and lower MF levels than unimodal and sensory periphery regions. In task states, the PLE increased and MF decreased while the variables' levels between core and periphery regions converged, respectively. A second dataset successfully replicated these observations. Additionally, a computational model supported that various degrees of extrinsic frequencies, mimicking inter‐trial intervals, can impact the PLE and MF levels. Our fMRI study demonstrated that the rest‐to‐task PLE modulation can dependent on the task's temporal structure, represented by the task's inter‐trial intervals. Albeit requiring further investigation and empirical evidence in future studies, this modulatory effect of the task's temporal structure offers the possibility to expand our horizon for understanding the brain‐environment interaction without neglecting other potential impacts on task‐related PLE changes. Albeit tentatively and in need of further neuroimaging analyses, our findings suggest a dynamically driven matching process between the brain and its context (environment), that is, temporo‐spatial alignment, which may play a role in consciousness (Northoff et al., 2020; Northoff & Huang, 2017; Northoff & Zilio, 2022).

## AUTHOR CONTRIBUTIONS

Philipp Klar provided data analysis, Figures, and manuscript writing. Yasir Çatal provided the IRASA method and surrogate simulation plus Figures. Robert Langner helped with manuscript editing. Zirui Huang helped with manuscript editing. Georg Northoff supervised and guided the analysis, helped with analysis design, analysis guidance, and manuscript writing.

## CONFLICT OF INTEREST

The authors declare no competing or conflict of interest.

## Supporting information


**Figure S1.** Inverse power‐law distributions and PLE where each line represents one subject. (a) SCP (row one) and JCP (row two) ROIs for time window 199–554. (b) SCP (row one) and JCP (row two) ROIs for time window 549–904. The PLE significantly increased and converged between core and periphery for the SCP and JCP ROIs in both task windows compared to the resting‐state. Vertical blue bars in the log–log power spectra represent the inter‐trial interval (52–60 s; 0.016–0.019 Hz). CV, coefficient of variation; PLE, power‐law exponent; SD, standard deviation.

**Figure S2.** Power spectra and MF where each line represents one subject. (a) SCP (row one) and JCP (row two) ROIs for time window 199–554. (b) SCP (row one) and JCP (row two) ROIs for time window 549–904. In both task windows, MF significantly decreased and converged between core and periphery for both the SCP and JCP ROIs compared to the resting‐state. Vertical pink bars in the power spectra represent the mean frequency and inter‐trial interval (52–60 s; 0.016–0.019 Hz) in blue. CV, coefficient of variation; MF, mean frequency; SD, standard deviation.

**Figure S3.** Voxel‐based PLE‐MF correlations in all ROIs. (a) SCP PLE‐MF correlations where task voxels (orange) are displayed on resting‐state voxels (green). (b) The second row displays the JCP PLE‐MF correlations.

**Figure S4.** Self‐ versus non‐self‐related PLE and MF time windows. (Left) Single‐trial 54 s time windows for the PLE. (Right) Single‐trial 54 s windows for MF.

**Figure S5.** Single ROI PLE and MF computations. (a) The SCP single ROI PLE values all shifted to higher values in task. (b) The SCP single ROI MF values accordingly shifted to slower frequencies in task (DAN, dorsal attention network; DMN, default‐mode network; FPN, fronto‐parietal network; SMN, somatomotor network; VAN, ventral attention network).

**Figure S6.** Correlation between the PLE in task states with the subjects' reaction times to trials in all ROIs. (a) The upper row displays the correlation between the PLE and the reaction times for self‐related trials in the SCP ROI (left) and JCP ROI (right). (b) The lower row shows the same correlation for non‐self‐related trials in the SCP ROI (left) and JCP ROI (right).

**Figure S7.** Inverse power‐law distributions and PLE where each line represents one subject. (a) SCP (row one) and JCP (row two) resting‐state. The core‐periphery comparison yielded significant PLE differences for both ROIs. (b) SCP (row one) and JCP (row two) task. In task states, the PLE significantly increased and converged between core and periphery regions for the SCP and JCP ROIs. The blue vertical bar represents the inter‐stimulus interval (ITI) range between 15.5–25.5 s (0.039–0.064 Hz). Vertical bars in the task log–log power spectra represent the inter‐trial interval (15.5–25.5 s; 0.039–0.064 Hz). CV, coefficient of variation; PLE, power‐law exponent; SD, standard deviation.

**Figure S8.** Power spectra and MF where each line represents one subject. (a) SCP (row one) and JCP (row two) resting‐state. The core‐periphery comparison yielded a significant MF difference for the SCP and JCP ROIs. (b) SCP (row one) and JCP (row two) task power spectra. In task states, the MF significantly decreased and converged between core and periphery regions for the SCP and JCP ROIs. Vertical bars in the power spectra represent the mean frequency and inter‐trial interval (15.5–25.5 s; 0.039–0.064 Hz). CV, coefficient of variation; MF, mean frequency; SD, standard deviation.

**Data S1.** Supporting Information.

**Table S1.** Core‐periphery comparison in two task time windows
**Table S2.** Voxel‐based PLE‐MF correlation
**Table S3.** Self‐ versus non‐self‐related trial time window results
**Table S4.** SCP single ROI PLE and MF results in resting‐state and task
**Table S5.** Correlation between BOLD PLE and estimated head motion PLE
**Table S6.** Correlation between task PLE and reaction times, power tests, and 95% confidence intervals
**Table S7.** PLE control analysis with surrogate data
**Table S8.** Core‐periphery comparison

## Data Availability

The datasets assessed in this fMRI analysis are available from the corresponding author upon reasonable request.
