# Supplementary figures and images for: Scale‐free dynamics of core‐periphery topography
Source: Hum Brain Mapp. 2022 Dec 29;44(5):1997–2017. doi: 10.1002/hbm.26187 (PMC9980897; doi:10.1002/hbm.26187)

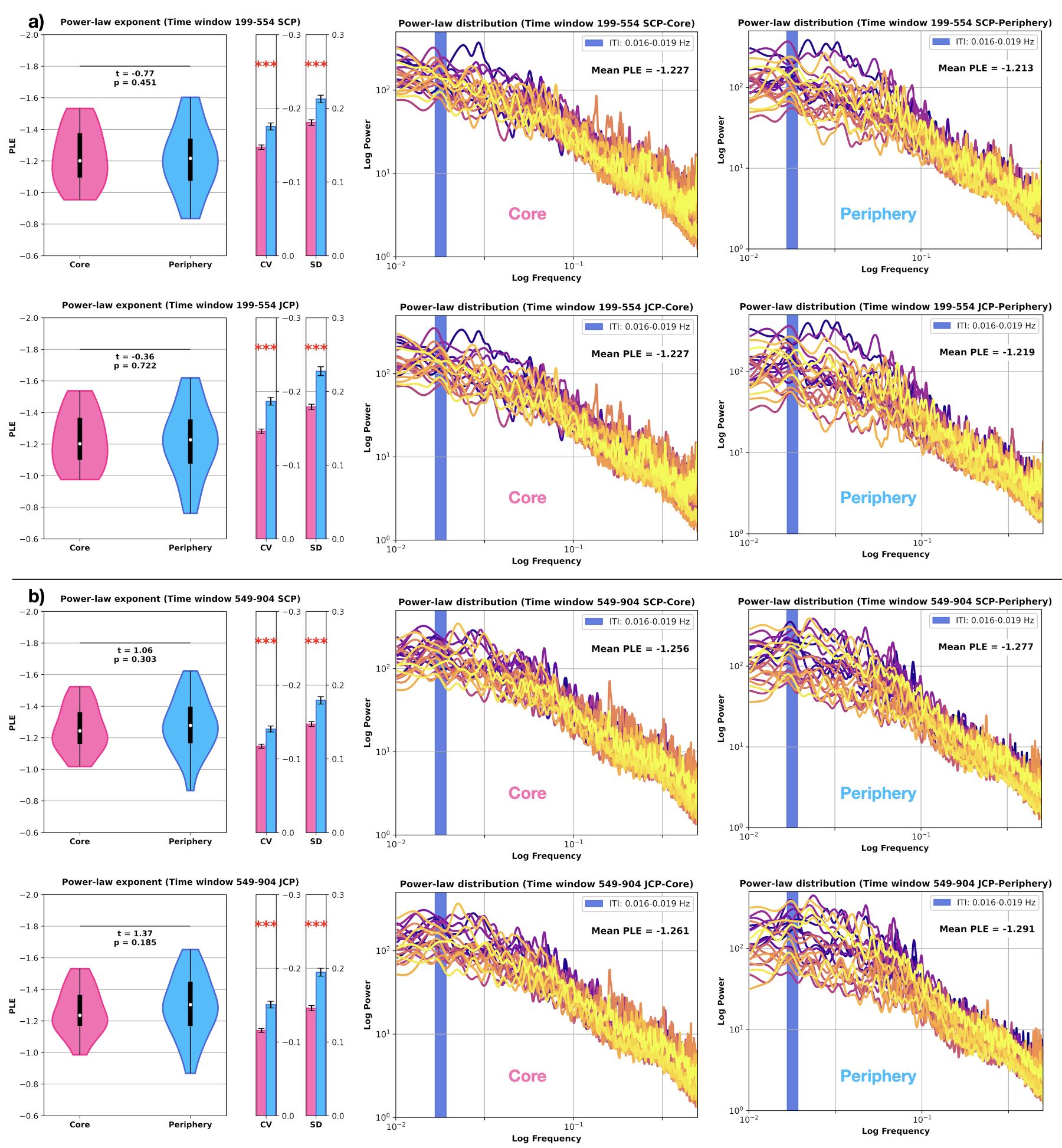

Supplement: Supplementary file 1 — Figure S1. Inverse power‐law distributions and PLE where each line represents one subject. (a) SCP (row one) and JCP (row two) ROIs for time window 199–554. (b) SCP (row one) and JCP (row two) ROIs for time window 549–904. The PLE significantly increased and converged between core and periphery for the SCP and JCP ROIs in both task windows compared to the resting‐state. Vertical blue bars in the log–log power spectra represent the inter‐trial interval (52–60 s; 0.016–0.019 Hz). CV, coefficient of variation; PLE, power‐law exponent; SD, standard deviation. [file HBM-44-1997-s003.tiff]

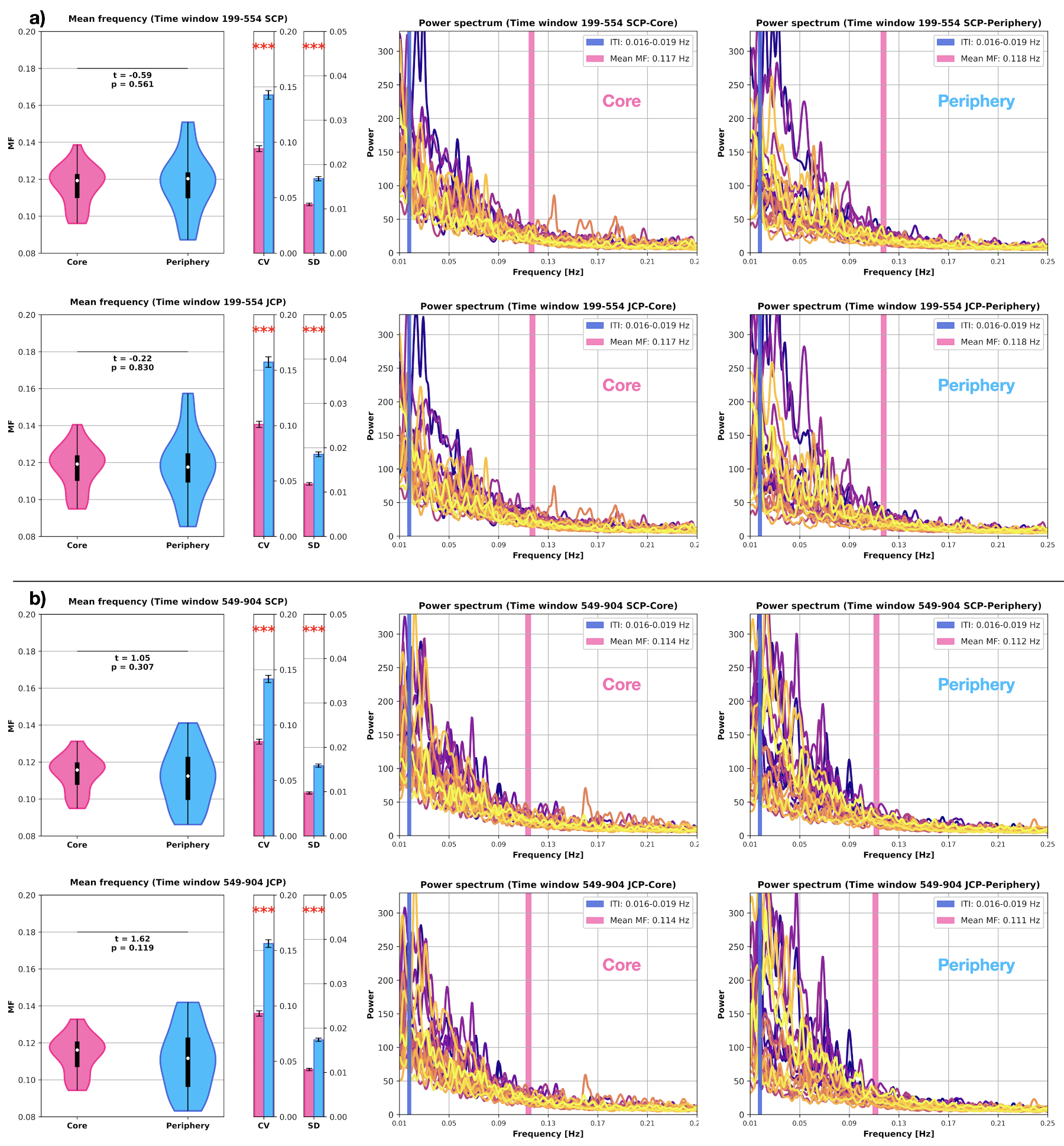

Supplement: Supplementary file 2 — Figure S2. Power spectra and MF where each line represents one subject. (a) SCP (row one) and JCP (row two) ROIs for time window 199–554. (b) SCP (row one) and JCP (row two) ROIs for time window 549–904. In both task windows, MF significantly decreased and converged between core and periphery for both the SCP and JCP ROIs compared to the resting‐state. Vertical pink bars in the power spectra represent the mean frequency and inter‐trial interval (52–60 s; 0.016–0.019 Hz) in blue. CV, coefficient of variation; MF, mean frequency; SD, standard deviation. [file HBM-44-1997-s010.tiff]

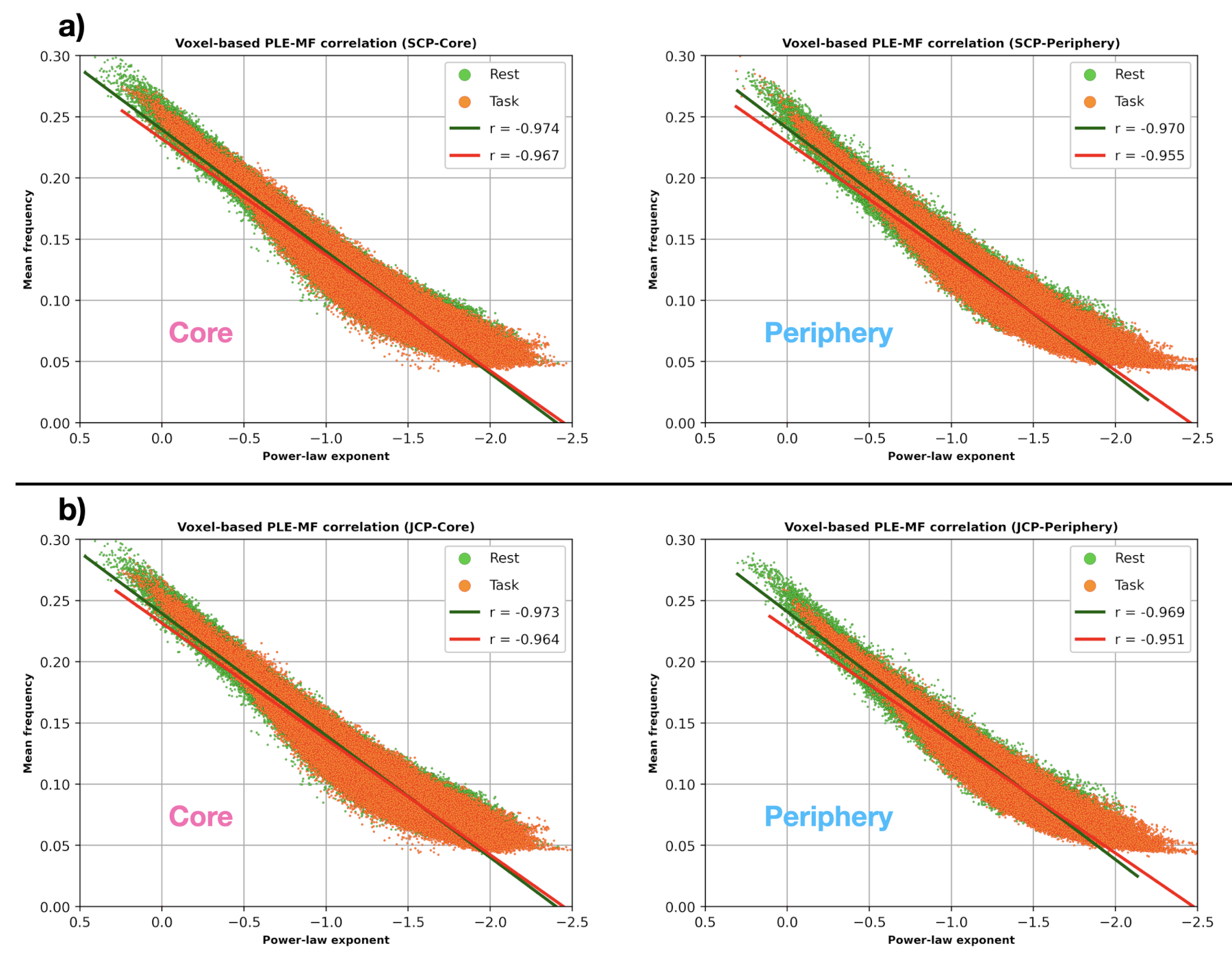

Supplement: Supplementary file 3 — Figure S3. Voxel‐based PLE‐MF correlations in all ROIs. (a) SCP PLE‐MF correlations where task voxels (orange) are displayed on resting‐state voxels (green). (b) The second row displays the JCP PLE‐MF correlations. [file HBM-44-1997-s008.tiff]

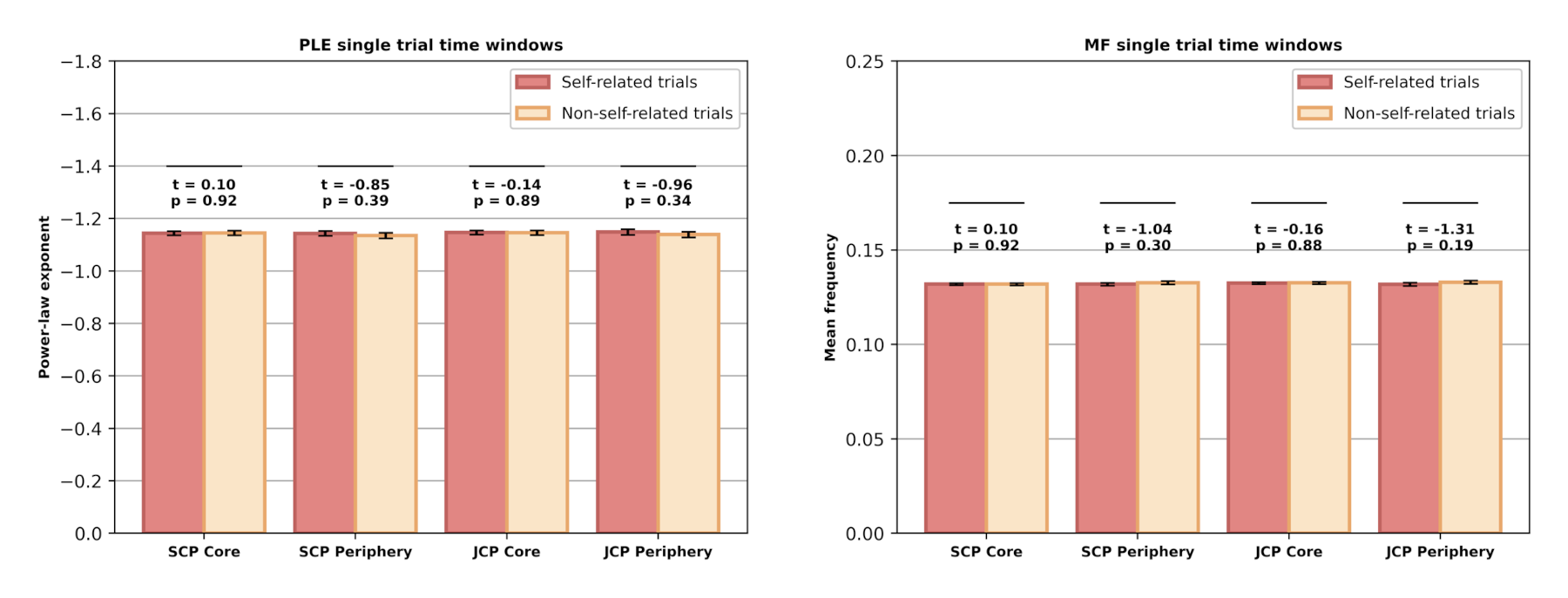

Supplement: Supplementary file 4 — Figure S4. Self‐ versus non‐self‐related PLE and MF time windows. (Left) Single‐trial 54 s time windows for the PLE. (Right) Single‐trial 54 s windows for MF. [file HBM-44-1997-s004.tiff]

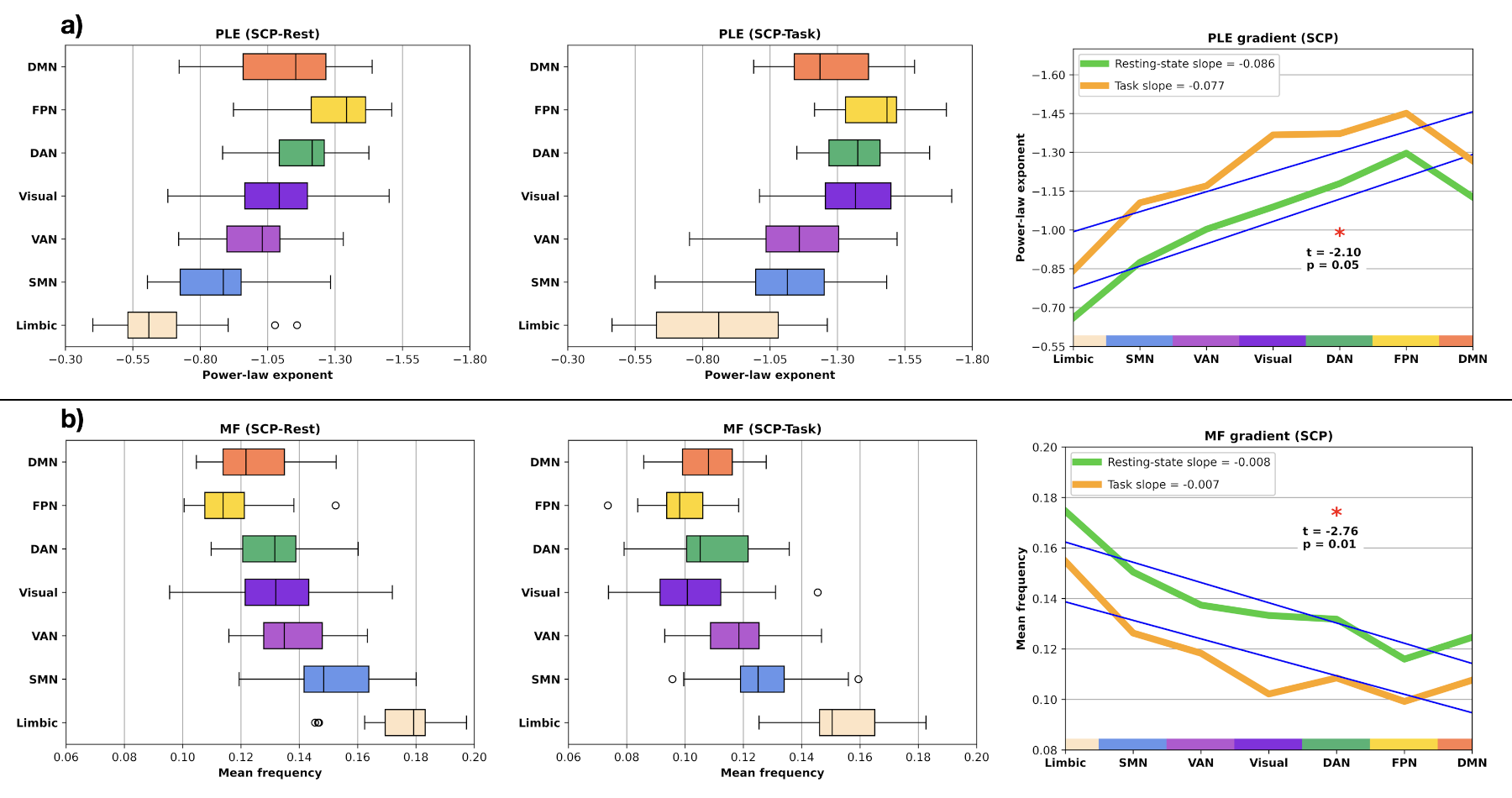

Supplement: Supplementary file 5 — Figure S5. Single ROI PLE and MF computations. (a) The SCP single ROI PLE values all shifted to higher values in task. (b) The SCP single ROI MF values accordingly shifted to slower frequencies in task (DAN, dorsal attention network; DMN, default‐mode network; FPN, fronto‐parietal network; SMN, somatomotor network; VAN, ventral attention network). [file HBM-44-1997-s005.tiff]

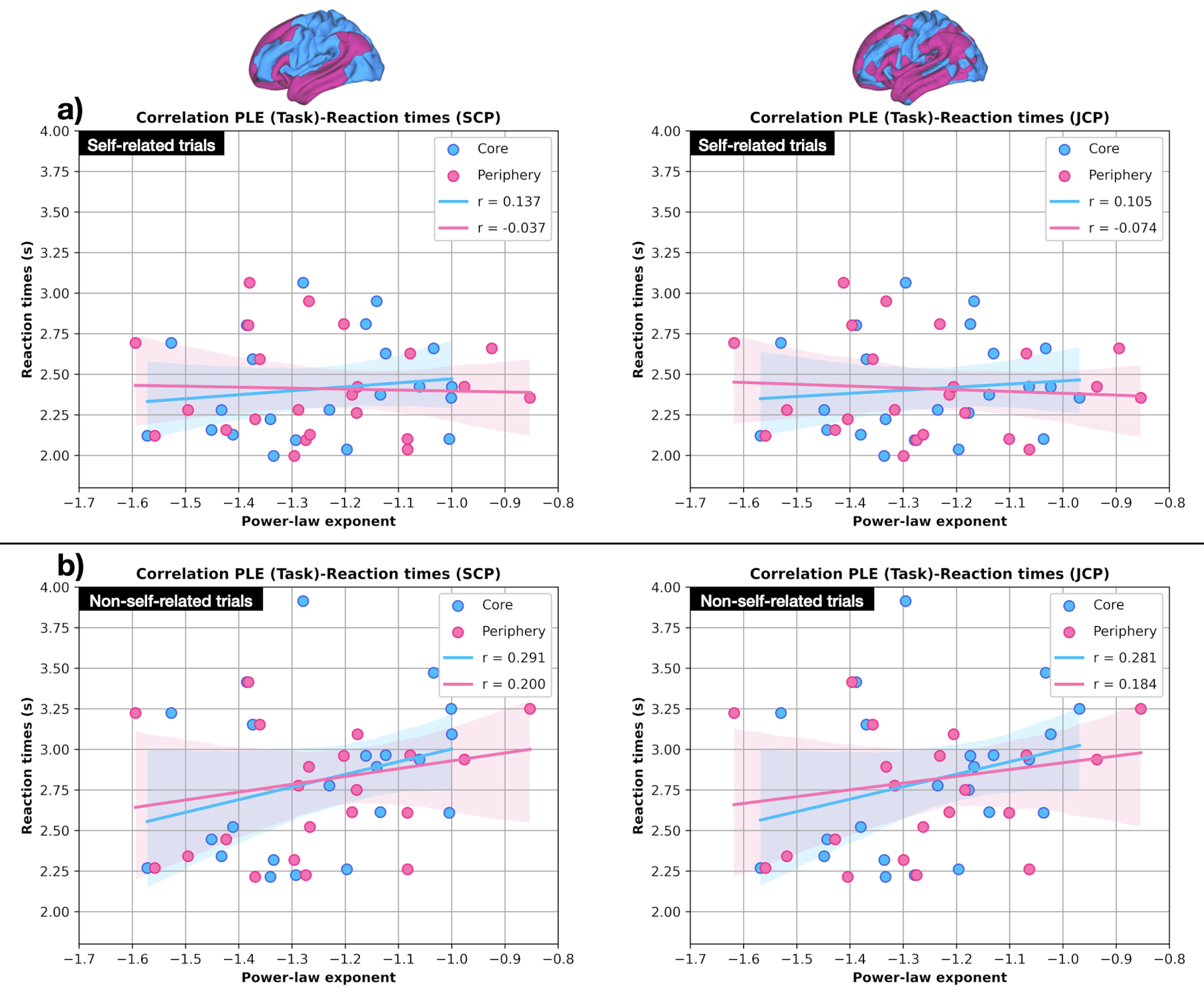

Supplement: Supplementary file 6 — Figure S6. Correlation between the PLE in task states with the subjects' reaction times to trials in all ROIs. (a) The upper row displays the correlation between the PLE and the reaction times for self‐related trials in the SCP ROI (left) and JCP ROI (right). (b) The lower row shows the same correlation for non‐self‐related trials in the SCP ROI (left) and JCP ROI (right). [file HBM-44-1997-s002.tiff]

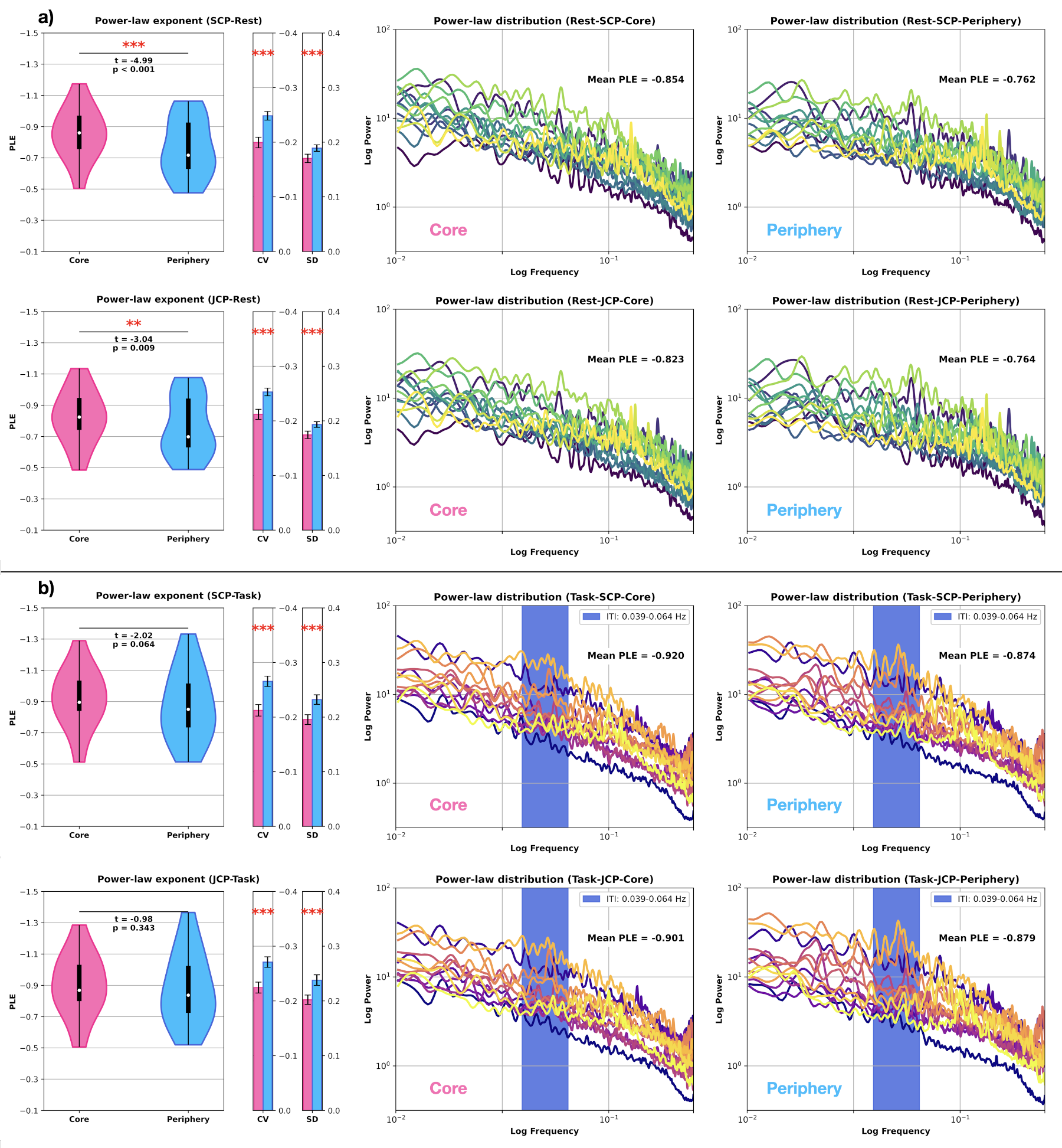

Supplement: Supplementary file 7 — Figure S7. Inverse power‐law distributions and PLE where each line represents one subject. (a) SCP (row one) and JCP (row two) resting‐state. The core‐periphery comparison yielded significant PLE differences for both ROIs. (b) SCP (row one) and JCP (row two) task. In task states, the PLE significantly increased and converged between core and periphery regions for the SCP and JCP ROIs. The blue vertical bar represents the inter‐stimulus interval (ITI) range between 15.5–25.5 s (0.039–0.064 Hz). Vertical bars in the task log–log power spectra represent the inter‐trial interval (15.5–25.5 s; 0.039–0.064 Hz). CV, coefficient of variation; PLE, power‐law exponent; SD, standard deviation. [file HBM-44-1997-s006.tiff]

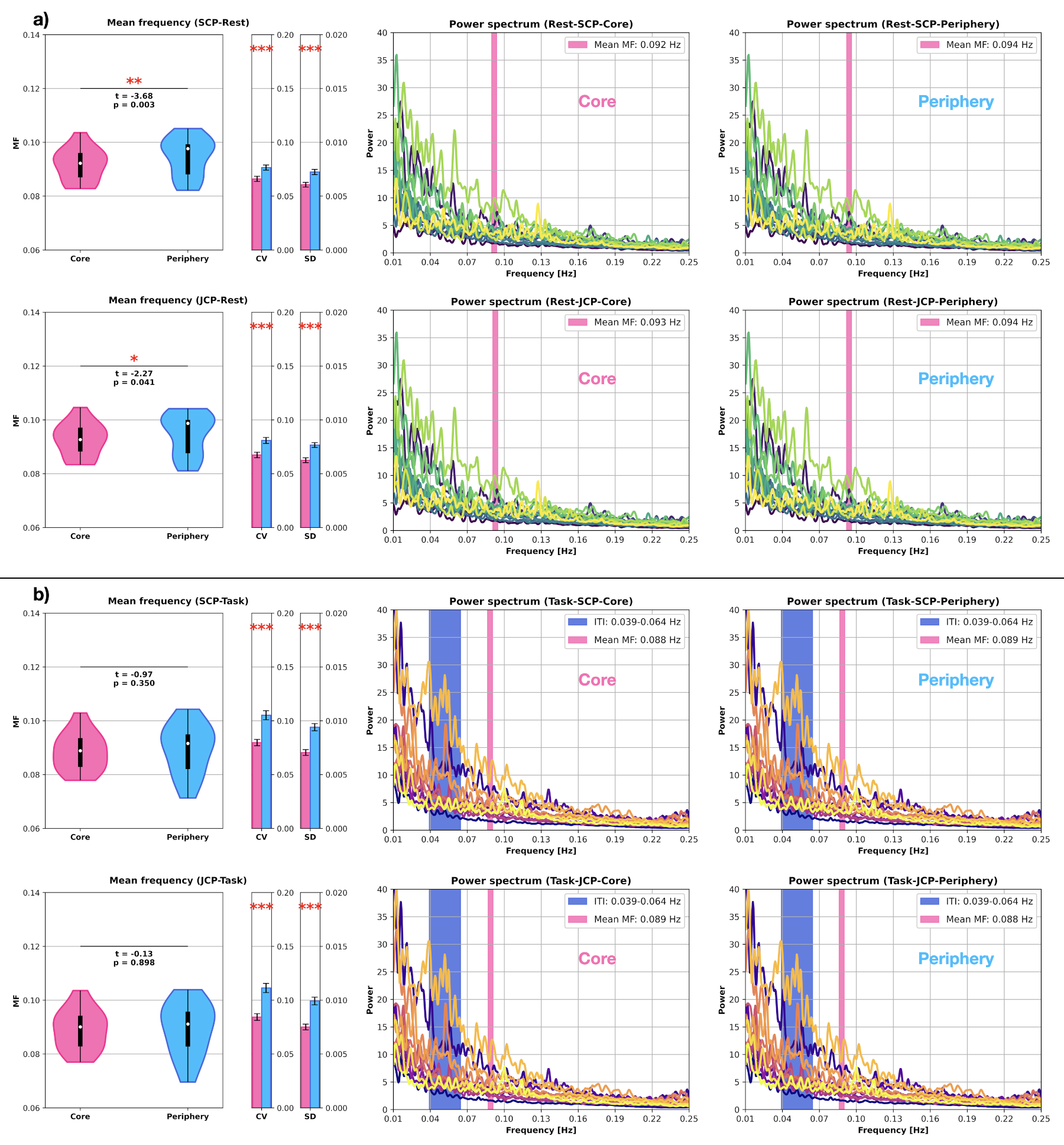

Supplement: Supplementary file 8 — Figure S8. Power spectra and MF where each line represents one subject. (a) SCP (row one) and JCP (row two) resting‐state. The core‐periphery comparison yielded a significant MF difference for the SCP and JCP ROIs. (b) SCP (row one) and JCP (row two) task power spectra. In task states, the MF significantly decreased and converged between core and periphery regions for the SCP and JCP ROIs. Vertical bars in the power spectra represent the mean frequency and inter‐trial interval (15.5–25.5 s; 0.039–0.064 Hz). CV, coefficient of variation; MF, mean frequency; SD, standard deviation. [file HBM-44-1997-s001.tiff]
