# Supplementary material for: Scale‐free dynamics of core‐periphery topography
Source: Hum Brain Mapp. 2022 Dec 29;44(5):1997–2017. doi: 10.1002/hbm.26187 (PMC9980897; doi:10.1002/hbm.26187)
Supplement: Supplementary file 9 — Data S1. Supporting Information. [file HBM-44-1997-s009.docx]

**Supplement for “Scale-free dynamics of core-periphery topography”**

**Overview of the replication and control analyses**

The functional magnetic resonance imaging (fMRI) analysis investigated the power-law exponent (PLE) and the mean frequency (MF) in the core-periphery topography of the human cerebral cortex. Our fMRI analysis applied control measurements in the primary dataset (Huang et al. 2017) and in a clinical replication dataset (Huang et al. 2018). The second replication dataset contained rest and task states with a comparable, yet shorter, task design with inter-trial intervals of 15.5-25.5 s (frequency range = 0.039-0.064 Hz) jittered in 2-second steps. Due to a similar task design, the second dataset provides a basis for replicating the primary dataset’s results. First, the supplement presents the various PLE and MF validation measurements assessed in our primary dataset (Huang et al. 2017) of our analysis. The results of Pearson’s and Spearman’s correlations between the BOLD’s PLE with the subjects’ reaction times follow the presentation of the primary dataset’s validation measurements. We then present the PLE control analysis of the comparison with surrogate data. Finally, we show the replication analysis of the second dataset (Huang et al. 2018).

**PLE control analyses in the primary dataset**

We computed a list of five control measurements to validate our PLE (and MF) findings observed in the primary dataset.

1. Two time windows (volumes 199-555 and 549-905) of the task time-series were matched to the resting-state length of 356 volumes. PLE and MF were computed for both windows to control that higher task PLE values were not a result of the task run’s longer length (compared to the resting-state run).
2. PLE and MF were computed and correlated on a voxel-based level for each subject in all ROIs, including rest and task states, to assess the systematic relationship between PLE and MF. The voxel-based PLE-MF analysis allowed checking wherever the ROI-based averages also hold and correlate on the voxel level in rest and task states, respectively.
3. We individually computed time windows for single trial PLE and MF (each window = 2 s trial plus 52 s post-stimulus activity) for self-related and non-self-related trials. Albeit in a limited frequency range, this allowed controlling if the task effect on PLE and MF holds irrespective of the task’s cognitive kind or content (self or non-self).
4. Several studies demonstrated variance of cortical features along a gradient, such as for functional and structural connectivity, cytoarchitecture, myeloarchitecture, gene expression, and the length (plus power) of the brain’s intrinsic neural timescales (Margulies et al. 2016; Baldassano et al. 2017; Huntenburg et al. 2018). We computed the PLE and MF for all single ROIs that constitute the SCP topography to control that PLE and MF changes in response to the task were manifest across the core-periphery topography.
5. Besides applying motion correction in the preprocessing, we extracted all six estimated head motion time-series (three translational and three rotational motion parameters). The head motion time-series were then transformed into the log-log frequency-domain to compute the head motion PLE. This allowed us to correlate the BOLD’s PLE for all ROIs in rest and task states with the estimated head motion PLE (Scalabrini et al. 2019) to exclude the possibility that BOLD PLE results were significantly affected by the subjects’ head motion during functional scanning.

**1. Two task time windows to control different lengths between rest and task runs**

We computed two time windows of the task’s time-series matched to the resting-state length of 356 volumes. Task window one contained volumes 199-554, and task window two volumes 549-904. We chose the volumes of both windows to each include seven trials, including the same number of four self-related and three non-self-related trials. We then analyzed the PLE and MF in both time windows. Both time windows showed the same PLE increases and MF decreases, as compared to the resting-state, as previously observed in the full-length task run. Figures 1 and 2, including Table 1, summarize the task windows’ results.

**Figure 1.** Inverse power-law distributions and PLE where each line represents one subject. **a)** SCP (row one) and JCP (row two) ROIs for time window 199-554. **b)** SCP (row one) and JCP (row two) ROIs for time window 549-904. The PLE significantly increased and converged between core and periphery for the SCP and JCP ROIs in both task windows compared to the resting-state. Vertical blue bars in the log-log power spectra represent the inter-trial interval (52-60 s; 0.016-0.019 Hz). PLE = power-law exponent, CV = Coefficient of variation, SD = Standard deviation.

**Figure 2.** Power spectra and MF where each line represents one subject. **a)** SCP (row one) and JCP (row two) ROIs for time window 199-554. **b)** SCP (row one) and JCP (row two) ROIs for time window 549-904. In both task windows, MF significantly decreased and converged between core and periphery for both the SCP and JCP ROIs compared to the resting-state. Vertical pink bars in the power spectra represent the mean frequency and inter-trial interval (52-60 s; 0.016-0.019 Hz) in blue. MF = mean frequency, CV = Coefficient of variation, SD = Standard deviation.

*Please insert Table 1 after or before Figure 2*

**2. Voxel-based correlation between PLE and MF**

We computed the PLE and MF on a voxel-based level in addition to the ROI-based results of our analysis. The voxel-based correlation between PLE and MF ensured that the connection between both variables, namely that increasing PLE values in task correlate with decreasing MF values and vice versa, hold on the smallest accessible level in addition to averaging voxels per ROI. The voxel-based PLE-MF correlations across all ROIs, including rest and task states, yielded very high correlations (r ≥ 0.951). Figure 3 displays the PLE-MF correlations summarized in Table 2.

**Figure 3.** Voxel-based PLE-MF correlations in all ROIs. **a)** SCP PLE-MF correlations where task voxels (orange) are displayed on resting-state voxels (green). **b)** The second row displays the JCP PLE-MF correlations.

*Please insert Table 2 after or before Figure 3*

**3. Self-related vs. non-self-related time windows**

We computed two types of trial time windows. Time window one only included self-related trials, whereas time window two included non-self-related trials. Each time window was constituted by the 2 s trial plus 52 s post-stimulus activity, providing a 54 s window. Albeit in a limited frequency range, the time windows allowed controlling if the task’s effect on PLE and MF holds irrespective of the task’s cognitive kind or content (self vs. non-self). Across all ROIs, neither the self-related nor the non-self-related trial windows yielded significant differences for PLE and MF. These non-significant results support that PLE increases and MF decreases in task states were not a product of the task’s cognitive kind or content. Figure 4 and Table 3 show the trial-window results for PLE and MF.

**Figure 4.** Self- vs. non-self-related PLE and MF time windows. **Left)** Single trial 54 s time windows for the PLE. **Right)** Single trial 54 s windows for MF.

*Please insert Table 3 after or before Figure 4*

**4. Single ROI PLE and MF analyses**

In addition to the PLE and MF measurements in the core-periphery topography, we investigated all single ROIs that constituted the SCP topography. The PLE and MF analyses in smaller individual ROIs allowed us to test that resting-state vs. task changes of both variables also hold on smaller spatial scales. We aimed to rule out the possibility that single constituting ROIs show a different PLE and MF behavior in rest and task states compared to the combined core-periphery topography. We observed a significant shift towards higher PLE and lower MF values (p < 0.001) when comparing the resting-state and task results across the single ROIs, hence ruling out the possibility that the PLE and MF behavior observed on the larger core-periphery scale does not hold on the smaller single ROI scale. Note that the median PLE in the visual ROI is relatively high, as previously observed in fMRI by He (2011) as well. Figure 5 and Table 4 summarize the single ROI results.

**Figure 5.** Single ROI PLE and MF computations. **a)** The SCP single ROI PLE values all shifted to higher values in task. **b)** The SCP single ROI MF values accordingly shifted to slower frequencies in task. (DMN = default-mode network; FPN = fronto-parietal network; DAN = dorsal attention network; VAN = ventral attention network; SMN = somatomotor network.)

*Please insert Table 4 after or before Figure 5*

**5. Controlling possible head motion effects on PLE analyses**

We applied motion correction in the fMRI preprocessing but additionally aimed to control that the subjects’ head motion during resting-state and task recordings did not substantially affect the BOLD’s PLE levels in the core-periphery topography. We extracted all six head motion time-series estimated by the preprocessing software AFNI (Cox 1996). The six estimated head motion time-series refer to six degrees of freedom for head motion during scanning: translational displacements (dS = displacement in the superior direction; dL = displacement in the left direction; and dP = displacement in the posterior direction) and rotational pitch, yaw, and roll. We individually transformed the head motion time-series into their log-log frequency-domain or power spectra. This allowed us to compute the PLE for each of the six head motion time-series for each subject. Finally, we calculated the Pearson correlation between BOLD PLE and head motion PLE for each ROI (SCP and JCP), head motion parameter (one to six), and rest vs. task states. The result comprises 24 correlations and p-values. We applied Bonferroni correction due to multiple comparisons (p = 0.05 threshold divided by four based on four ROIs per head motion parameter). The resulting statistical threshold after the Bonferroni correction is p = 0.0125. Assessing the correlation between the BOLD’s PLE and the estimated head motion parameters’ PLE allowed us to check and exclude the possibility that BOLD PLE levels were significantly affected by the subjects’ head motion during functional scanning (Scalabrini et al. 2019). Table 5 shows the correlation results between the BOLD and head motion PLE.

*Please insert Table 5 here*

**Correlation between task PLE and the subjects’ reaction times**

Aim three of our analysis investigated the possibility that the task’s infra-slow frequency range (0.016-0.019 Hz), in addition to other possible effects, modulated the PLE increases in task states compared to the resting-state. We correlated the task’s PLE for all SCP and JCP core and periphery regions with the subjects’ reaction times (RT) in response to the trials. More precisely, we computed eight PLE-RT correlations using Pearson’s r and eight PLE-RT correlations using Spearman’s rho as follows:

1. The SCP and JCP core and periphery regions’ task PLE values were correlated with the subjects’ self- and non-self-related trials using Pearson correlation, yielding eight PLE-RT correlations.
2. We repeated the same PLE-RT correlations as in (1) using Spearman’s rho instead of Pearson’s r, yielding a second set of eight PLE-RT correlations.

We applied the Pearson and Spearman correlations individually for self- and non-self-related trials with all ROI-based PLE values to test wherever task PLE levels significantly correlated with the subjects’ reaction times in response to the task’s trials. We also report two-sided 95% confidence intervals (2.5% - 97.5%) for Pearson’s r and Spearman’s rho correlations. We applied 599 bootstrap replications and the BCa method for the Spearman confidence intervals.

Furthermore, we ran power tests to assess the probability that the PLE-RT correlations reject a false null hypothesis, i.e., that the PLE-RT results will not make a Type II error. In our case, the Type II error is the probability of failing to reject a non-significant PLE-RT correlation (null hypothesis) while the PLE-RT correlation is significant (alternative hypothesis). The power tests applied N = 23 (number of subjects), r = the respective Pearson’s r or Spearman’s rho, and the significance level = 0.05. The power test results are equal to 1 minus the Type II error probability.

All Pearson’s correlation coefficients and all Spearman’s rho correlation results turned out non-significant (Figure 6 and Table 6). At the same time, the 95% confidence intervals showed a wide range, and the power tests yielded low results. In addition, the power tests generally tended to be higher for non-self-related than for self-related trials. Together, these results implicate a rather complex interaction between the task-related PLE levels and the subjects’ reaction times. Specifically designed task paradigms that systematically vary the trials’ ITI are required for a more in-depth investigation wherever self- vs. non-self-related trial types individually modulate task-related PLE changes.

**Figure 6.** Correlation between the PLE in task states with the subjects’ reaction times to trials in all ROIs. **a)** The upper row displays the correlation between the PLE and the reaction times for self-related trials in the SCP ROI (left) and JCP ROI (right). **b)** The lower row shows the same correlation for non-self-related trials in the SCP ROI (left) and JCP ROI (right).

*Please insert Table 6 after or before Figure 6*

**PLE control analysis: comparison with surrogate data**

In addition to the IRASA method, we further tested the suggested scale-free dynamics of our data by comparing the goodness of fit of the power-law to the PSDs of real data and simulated fGN (Clauset et al. 2009; He 2011; Tagliazucchi et al. 2013; Scalabrini et al. 2017; Çatal et al. 2022). All ROIs had P-values, that is, the fraction of synthetic time series that had a worse fit than real data, were bigger than 0.1, except SCP Periphery in Task which was 0.057. Table 7 displays the results.

*Please insert Table 7 here*

**Replication dataset**

Besides performing PLE and MF control analyses in the primary dataset, we assessed a replication dataset. The replication dataset re-used clinical data (Huang et al. 2018) from 20 right-handed adults (male/female: 8/12; age 32-64 years). Subjects were undergoing an elective transsphenoidal approach for the resection of a pituitary microadenoma. We excluded six subjects from the fMRI analyses based on excessive head motion during scanning. Like our primary dataset, the replication dataset used a sparse event-related design with 60 trials. These trials included 30 own and 30 other (an unknown person’s name) names delivered in a pseudo-random order. These names were recorded by a familiar voice from one of the patient’s family members with an audio clip lasting for 0.5 s. The unknown names were individually matched to each patient’s name by gender and number of syllables. Inter-trial intervals (ITIs) ranged from 15.5 to 25.5 s (frequency range = 0.039-0.064 Hz) jittered in 2 second steps. We analyzed the PLE and MF in rest and task states. In accordance with the results obtained in the primary dataset, we observed increasing PLE and decreasing MF values in task states (compared to the resting-state) that, like in the primary dataset, also converged between the core and periphery.

**PLE in resting-state**

The PLE values of both core regions (SCP = -0.854; JCP = -0.823) were significantly higher than in the respective periphery regions (SCP = -0.762, t = -4.99, p < 0.001; JCP = -0.764, t = -3.04, p = 0.009). The CV was significantly higher (p < 0.001) in the periphery regions for both ROIs (SCP = -0.248; JCP = -0.253) than for the core regions (SCP = -0.199; JCP = -0.212).

**PLE in task**

Significant core-periphery comparisons, as observed in the resting-state, completely vanished during task states. The PLE values between core and periphery converged for both ROIs (SCP: t = -2.02, p = 0.064; JCP: t = -0.98, p = 0.343). Compared to the resting-state, the core PLE (SCP = -0.92; JCP = -0.901) and periphery PLE (SCP = -0.874; JCP = -0.879) significantly increased (p < 0.001). The CV was significantly higher (p < 0.001) in the periphery regions for both ROIs (SCP = -0.265; JCP = -0.271) than for the core regions (SCP = -0.212; JCP = -0.224). Figure 7 displays the resting-state and task power-laws and PLE.

**Figure 7.** Inverse power-law distributions and PLE where each line represents one subject. **a)** SCP (row one) and JCP (row two) resting-state. The core-periphery comparison yielded significant PLE differences for both ROIs. **b)** SCP (row one) and JCP (row two) task. In task states, the PLE significantly increased and converged between core and periphery regions for the SCP and JCP ROIs. The blue vertical bar represents the inter-stimulus interval (ITI) range between 15.5-25.5 s (0.039-0.064 Hz). Vertical bars in the task log-log power spectra represent the inter-trial interval (15.5-25.5 s; 0.039-0.064 Hz). PLE = power-law exponent, CV = Coefficient of variation, SD = Standard deviation.

**MF in resting-state**

The MF in the SCP periphery region (MF = 0.094) was significantly higher (t = -3.68, p = 0.003) than in the core region (MF = 0.092). Likewise, the MF in the JCP periphery region (MF = 0.094 Hz) was significantly higher (t = -2.27, p = 0.041) than in the core region (MF = 0.093 Hz). The CV values were always higher in the periphery regions (p < 0.001). Inter-subject CV of MF values showed the same pattern as the CV of PLE, being significantly higher (p < 0.001) in the periphery regions (SCP = 0.0765; JCP = 0.123) compared to the corresponding core regions (SCP = 0.0765; JCP = 0.081).

**MF in task**

In contrast to the resting-state, the core-periphery comparison no longer yielded a significant difference in task states. Both MF core (SCP = 0.088; JCP = 0.089) and MF periphery (SCP = 0.089; JCP = 0.088) decreased to the same level, respectively (SCP: t = -0.97, p = 0.35; JCP: t = -0.13, p = 0.898). The CV was significantly higher (p < 0.001) in the periphery regions for both ROIs (SCP = 0.1051; JCP = 0.111) than for the core regions (SCP = 0.079; JCP = 0.084). MF resting-state and task results are displayed in Figure 8 and Table 8 provide an overview of both resting-state/task PLE/MF results.

**Figure 8.** Power spectra and MF where each line represents one subject. **a)** SCP (row one) and JCP (row two) resting-state. The core-periphery comparison yielded a significant MF difference for the SCP and JCP ROIs. **b)** SCP (row one) and JCP (row two) task power spectra. In task states, the MF significantly decreased and converged between core and periphery regions for the SCP and JCP ROIs. Vertical bars in the power spectra represent the mean frequency and inter-trial interval (15.5-25.5 s; 0.039-0.064 Hz). MF = mean frequency, CV = Coefficient of variation, SD = Standard deviation.

*Please insert Table 8 here*

**References**

Cox RW. 1996. AFNI: software for analysis and visualization of functional magnetic resonance neuroimages. *Comput. Biomed. Res.* 29(3):162-173. <https://doi.org/10.1006/cbmr.1996.0014>

Catal Y, Gomez-Pilar J, Northoff G. 2022. Intrinsic Dynamics and Topography of Sensory Input Systems. Cereb. Cortex. <https://doi.org/10.1093/cercor/bhab504>

Clauset A, Shalizi CR, Newman MEJ. 2009. Power-law distributions in empirical data. *SIAM Rev.* 51(4):661-703. [https://doi.org/10.1137/07071011](https://doi.org/10.1137/070710111)

He B. 2011. Scale-free properties of the functional magnetic resonance imaging signal during rest and task. *J Neurosci.* 31(39):13786–13795. <https://doi.org/10.1523/JNEUROSCI.2111-11.2011>

Huang Z, Zhang J, Longtin A, Dumont G, Duncan NW, Pokorny J, Qin P, Dai R, Ferri F, Weng X, Northoff G. 2017. Is There a Nonadditive Interaction Between Spontaneous and Evoked Activity? Phase-Dependence and Its Relation to the Temporal Structure of Scale-Free Brain Activity. *Cereb. Cortex.* 27:1037-1059. <https://doi.org/10.1093/cercor/bhv288>

Huang Z, Zhang J, Wu J, Liu X, Xu J, Zhang J, Qin P, Dai R, Yang Z, Mao Y, Hudetz AG, Northoff G. 2018. Disrupted neural variability during propofol-induced sedation and unconsciousness. *Hum. Brain Mapp.* 39(11):4533–4544. [https://dx.doi.org/10.1002%2Fhbm.24304](https://dx.doi.org/10.1002%252525252Fhbm.24304)

Scalabrini A, Huang Z, Mucci C, Perrucci MG, Ferretti A, Fossati A, Romani GL, Northoff G, Ebisch SJH. 2017. How spontaneous brain activity and narcissistic features shape social interaction. *Sci Rep.* 7(1):9986. <https://doi.org/10.1038/s41598-017-10389-9>

Scalabrini A, Ebisch SJH, Huang Z, Di Plinio S, Perrucci MG, Romani GL, Mucci C, Northoff G. 2019. Spontaneous Brain Activity Predicts Task-Evoked Activity During Animate Versus Inanimate Touch. *Cereb. Cortex.* 29(11):4628-4645. <https://doi.org/10.1093/cercor/bhy340>

Tagliazucchi E, von Wegner F, Morzelewski A, Brodbeck V, Jahnke K, Laufs H. 2013. Breakdown of long-range temporal dependence in default mode and attention networks during deep sleep. *Proc. Natl. Acad. Sci. U.S.A.* 110(38):15419–15424. <https://doi.org/10.1073/pnas.1312848110>

Scalabrini A, Huang Z, Mucci C, Perrucci MG, Ferretti A, Fossati A, Romani GL, Northoff G, Ebisch SJH. 2017. How spontaneous brain activity and narcissistic features shape social interaction. *Sci Rep.* 7(1):9986. <https://doi.org/10.1038/s41598-017-10389-9>

Scalabrini A, Ebisch SJH, Huang Z, Di Plinio S, Perrucci MG, Romani GL, Mucci C, Northoff G. 2019. Spontaneous Brain Activity Predicts Task-Evoked Activity During Animate Versus Inanimate Touch. *Cereb. Cortex.* 29(11):4628-4645. <https://doi.org/10.1093/cercor/bhy340>
