# Supplementary material for: Scale‐free dynamics of core‐periphery topography
Source: Hum Brain Mapp. 2022 Dec 29;44(5):1997–2017. doi: 10.1002/hbm.26187 (PMC9980897; doi:10.1002/hbm.26187)
Supplement: Supplementary file 10 — Table S1. Core‐periphery comparison in two task time windows Table S2. Voxel‐based PLE‐MF correlation Table S3. Self‐ versus non‐self‐related trial time window results Table S4. SCP single ROI PLE and MF results in resting‐state and task Table S5. Correlation between BOLD PLE and estimated head motion PLE Table S6. Correlation between task PLE and reaction times, power tests, and 95% confidence intervals Table S7. PLE control analysis with surrogate data Table S8. Core‐periphery comparison [file HBM-44-1997-s007.docx]

**Tables for supplement:**

| **Table 1**  Core-Periphery Comparison in two task time windows | | | | |  | |
| --- | --- | --- | --- | --- | --- | --- |
| Computation | Time window | ROI | Core | Periphery | t-value | p-value |
| PLE | 199-554 | SCP | -1.228  (-0.141 / 0.176) | -1.213  (-0.172 / 0.212) | -0.77 | 0.451 |
|  |  | JCP | -1.227  (-0.146 / 0.179) | -1.219  (0.187 / -0.227) | -0.36 | 0.722 |
|  | 549-904 | SCP | -1.256  (-0.115 / 0.147) | -1.277  (-0.133 / 0.173) | 1.06 | 0.303 |
|  |  | JCP | -1.261  (-0.116 / 0.146) | -1.291  (-0.151 / 0.195) | 1.37 | 0.185 |
| MF | 199-554 | SCP | 0.117  (0.105 / 0.012) | 0.118  (0.148 / 0.017) | -0.59 | 0.561 |
|  |  | JCP | 0.117  (0.101 / 0.011) | 0.118  (0.157 / 0.019) | -0.22 | 0.830 |
|  | 549-904 | SCP | 0.114  (0.095 / 0.011) | 0.112  (0.141 / 0.011) | 1.05 | 0.307 |
|  |  | JCP | 0.114  (0.093 / 0.01) | 0.111  (0.156 / 0.017) | 1.62 | 0.119 |
| Data represents mean values including their respective coefficient of variation (CV) (first value or left in bracket) and standard deviation (SD) (second or right value in bracket). PLE = power-law exponent; MF = mean frequency; statistics = Student’s paired t-Test. | | | | | | |

| **Table 2**  Voxel-based PLE-MF correlation | | | |  |
| --- | --- | --- | --- | --- |
| Computations | ROIs | Rest | Task | p-value |
| Pearson’s r | SCP Core | -0.974 | -0.967 | 0 |
|  | SCP Periphery | -0.970 | -0.955 | 0 |
|  | JCP Core | -0.973 | -0.964 | 0 |
|  | JCP Periphery | -0.969 | -0.951 | 0 |
| PLE-MF correlations are based on voxel-based data. p-values are 0 due to > 5.000.000 voxels for PLE and MF, respectively. | | | | |

| **Table 3**  Self- vs. non-self-related trial time window results | | | |  | |
| --- | --- | --- | --- | --- | --- |
| Computations | ROI | Self-related | Non-self-related | t-value | p-value |
| PLE | SCP Core | -1.144 | -1.145 | 0.10 | 0.923 |
|  | SCP Periphery | -1.143 | -1.135 | -0.85 | 0.394 |
|  | JCP Core | -1.147 | -1.146 | -0.14 | 0.888 |
|  | JCP Periphery | -1.149 | -1.139 | -0.96 | 0.338 |
| MF | SCP Core | 0.132 | 0.132 | 0.10 | 0.918 |
|  | SCP Periphery | 0.132 | 0.133 | -1.04 | 0.301 |
|  | JCP Core | 0.132 | 0.133 | -0.16 | 0.875 |
|  | JCP Periphery | 0.132 | 0.133 | -1.31 | 0.191 |
| Data represents mean values. Statistics = Student’s paired t-Test. | | | | | |

| **Table 4**  SCP single ROI PLE and MF results in resting-state and task | | | |  | |
| --- | --- | --- | --- | --- | --- |
| Computations | ROI | Rest | Task | t-value | p-value |
| PLE | Visual | -1.089 | -1.368 | 7.90 | p < 0.001 |
|  | SMN | -0.875 | -1.105 | 6.5411 | p < 0.001 |
|  | DAN | -1.179 | -1.372 | 7.9459 | p < 0.001 |
|  | VAN | -1.003 | -1.170 | 4.8937 | p < 0.001 |
|  | Limbic | -0.660 | -0.843 | 4.5189 | p < 0.001 |
|  | FPN | -1.297 | -1.451 | 7.7406 | p < 0.001 |
|  | DMN | -1.126 | -1.267 | 4.9655 | p < 0.001 |
| MF | Visual | 0.1332 | 0.1021 | 8.4457 | p < 0.001 |
|  | SMN | 0.1504 | 0.1263 | 7.3379 | p < 0.001 |
|  | DAN | 0.1318 | 0.1085 | 8.2545 | p < 0.001 |
|  | VAN | 0.1374 | 0.1183 | 7.1547 | p < 0.001 |
|  | Limbic | 0.1746 | 0.1547 | 8.0194 | p < 0.001 |
|  | FPN | 0.1159 | 0.0992 | 8.9261 | p < 0.001 |
|  | DMN | 0.1246 | 0.1076 | 8.0094 | p < 0.001 |
| Data represents mean values. Statistics = Student’s paired t-Test between rest and task states. (DMN = default-mode network; FPN = fronto-parietal network; DAN = dorsal attention network; VAN = ventral attention network; SMN = somatomotor network.) | | | | | |

| **Table 5**  Correlation between BOLD PLE and estimated head motion PLE | | | |  |  |
| --- | --- | --- | --- | --- | --- |
| Head motion parameter | ROI | Pearson’s r | p-value | Pearson’s r | p-value |
|  |  | Rest | | Task | |
| **Rotation** |  |  |  |  |  |
| Roll | SCP Core | 0.315 | 0.143 | 0.256 | 0.238 |
|  | SCP Periphery | 0.19 | 0.387 | 0.143 | 0.514 |
|  | JCP Core | 0.31 | 0.146 | 0.243 | 0.264 |
|  | JCP Periphery | 0.16 | 0.462 | 0.099 | 0.653 |
| Pitch | SCP Core | 0.42 | 0.048 | 0.261 | 0.230 |
|  | SCP Periphery | 0.30 | 0.160 | 0.165 | 0.451 |
|  | JCP Core | 0.40 | 0.059 | 0.238 | 0.274 |
|  | JCP Periphery | 0.30 | 0.172 | 0.145 | 0.509 |
| Yaw | SCP Core | 0.63 | 0.001 | 0.261 | 0.230 |
|  | SCP Periphery | 0.52 | 0.012 | 0.219 | 0.315 |
|  | JCP Core | 0.62 | 0.001 | 0.257 | 0.237 |
|  | JCP Periphery | 0.49 | 0.019 | 0.185 | 0.399 |
| **Translation** |  |  |  |  |  |
| dS | SCP Core | 0.43 | 0.040 | 0.048 | 0.829 |
|  | SCP Periphery | 0.36 | 0.087 | -0.059 | 0.788 |
|  | JCP Core | 0.44 | 0.037 | 0.044 | 0.843 |
|  | JCP Periphery | 0.33 | 0.126 | -0.085 | 0.700 |
| dL | SCP Core | 0.48 | 0.019 | 0.090 | 0.684 |
|  | SCP Periphery | 0.35 | 0.098 | 0.051 | 0.819 |
|  | JCP Core | 0.50 | 0.016 | 0.098 | 0.658 |
|  | JCP Periphery | 0.30 | 0.161 | 0.026 | 0.906 |
| dP | SCP Core | 0.54 | 0.008 | 0.307 | 0.155 |
|  | SCP Periphery | 0.49 | 0.019 | 0.401 | 0.058 |
|  | JCP Core | **0.54** | **0.008** | 0.329 | 0.126 |
|  | JCP Periphery | 0.48 | 0.020 | 0.420 | 0.046 |
| Pearson’s correlation between ROI-based BOLD PLE and estimated head motion PLE. Bonferroni correction (p = 0.05 threshold divided by four based on four ROIs per head motion parameter). Hence, the chosen threshold for statistical significance after the Bonferroni correction is p = 0.0125. Significant correlations (p ≤ 0.0125) are printed bold. (dS = displacement in the superior direction, dL = displacement in the left direction, dP = displacement in the posterior direction.) | | | | | |

| **Table 6**  Correlation between task PLE and reaction times, power tests, and 95% confidence intervals | | | | |  |
| --- | --- | --- | --- | --- | --- |
|  | SCP Core | SCP Periphery | JCP Core | JCP Periphery | Mean correlation across ROIs |
| **Self-related trials** |  |  |  |  |  |
| Pearson’s r | 0.137 | -0.037 | 0.105 | -0.074 | 0.033 |
| t-value | 0.632 | -0.168 | 0.486 | -0.338 |  |
| p-value | 0.534 | 0.8678 | 0.632 | 0.7386 |  |
| Power test | 0.095 | 0.053 | 0.076 | 0.062 |  |
| 95% confidence interval | -0.29 - 0.52 | -0.44 - 0.38 | -0.32 - 0.49 | -0.47 - 0.35 |  |
| Spearman’s ρ | 0.201 | -0.001 | 0.183 | -0.072 | 0.078 |
| S-value | 1618 | 2026 | 1654 | 2170 |  |
| p-value | 0.357 | 0.998 | 0.402 | 0.743 |  |
| Power test | 0.152 | 0.049 | 0.134 | 0.062 |  |
| 95% confidence interval | -0.24 - 0.52 | -0.49 - 0.38 | -0.33 - 0.50 | -0.49 - 0.35 |  |
| **Non-self-related trials** |  |  |  |  |  |
| Pearson’s r | 0.291 | 0.200 | 0.281 | 0.184 | 0.239 |
| t-value | 1.395 | 0.936 | 1.342 | 0.857 |  |
| p-value | 0.1775 | 0.36 | 0.1938 | 0.401 |  |
| Power test | 0.277 | 0.151 | 0.260 | 0.135 |  |
| 95% confidence interval | -0.14 - 0.63 | -0.23 - 0.57 | -0.15 - 0.62 | -0.25 - 0.55 |  |
| Spearman’s ρ | 0.343 | 0.202 | 0.322 | 0.174 | 0.260 |
| S-value | 1330 | 1616 | 1372 | 1672 |  |
| p-value | 0.110 | 0.355 | 0.134 | 0.426 |  |
| Power test | 0.370 | 0.153 | 0.331 | 0.125 |  |
| 95% confidence interval | -0.11 - 0.69 | -0.26 - 0.68 | -0.17 - 0.71 | -0.35 - 0.61 |  |
| Corrections for multiple comparisons were not applied. Power tests included N = 23, r = respective Pearson’s r or Spearman’s rho, significance level = 0.05. We applied 599 bootstrap replications and the bca method for the Spearman confidence intervals. | | | | | |

| **Table 7**  PLE control analysis with surrogate data | |
| --- | --- |
| ROI | p-value |
| Rest SCP Core | 0.237 |
| Rest SCP Periphery | 0.202 |
| Rest JCP Core | 0.651 |
| Rest JCP Periphery | 0.563 |
| Task SCP Core | 0.403 |
| Task SCP Periphery | 0.057 |
| Task JCP Core | 0.178 |
| Task JCP Periphery | 0.196 |

| **Table 8**  Core-Periphery Comparison | | | | |  | |
| --- | --- | --- | --- | --- | --- | --- |
| Computations | Session | ROI | Core | Periphery | t-value | p-value |
| PLE | Rest | SCP | -0.854  (-0.192 / 0.17) | -0.771  (-0.241 / 0.189) | -499.00 | p < 0.001 |
|  |  | JCP | -0.824  (-0.204 / 0.174) | -0.776  (-0.247 / 0.193) | -3.04 | 0.009 |
|  | Task | SCP | -0.92  (-0.205 / 0.196) | -0.888  (-0.259 / 0.232) | -2.02 | 0.064 |
|  |  | JCP | -0.903  (-0.216 / 0.202) | -0.895  (-0.266 / 0.238) | -0.98 | 0.343 |
| MF | Rest | SCP | 0.092  (0.064 / 0.006) | 0.094  (0.076 / 0.007) | -3.68 | 0.003 |
|  |  | JCP | 0.093  (0.065 / 0.006) | 0.094  (0.082 / 0.008) | -2.27 | 0.041 |
|  | Task | SCP | 0.0885  (0.077 / 0.007) | 0.089  (0.108 / 0.009) | -0.97 | 0.350 |
|  |  | JCP | 0.0889  (0.081 / 0.007) | 0.088 (0.117 / 0.009) | -0.13 | 0.898 |
| Data represents mean values including their respective coefficient of variation (CV) (first value or left in bracket) and standard deviation (SD) (second or right value in bracket). PLE = power-law exponent; MF = mean frequency; statistics = Student’s paired t-Test. | | | | | | |
